# Salivary miRNA Expression in Children With Persistent Post-concussive Symptoms

**DOI:** 10.3389/fpubh.2022.890420

**Published:** 2022-05-30

**Authors:** Katherine E. Miller, James P. MacDonald, Lindsay Sullivan, Lakshmi Prakruthi Rao Venkata, Junxin Shi, Keith Owen Yeates, Su Chen, Enas Alshaikh, H. Gerry Taylor, Amanda Hautmann, Nicole Asa, Daniel M. Cohen, Thomas L. Pommering, Elaine R. Mardis, Jingzhen Yang, the NCH Concussion Research Group

**Affiliations:** ^1^The Steve and Cindy Rasmussen Institute for Genomic Medicine, Abigail Wexner Research Institute at Nationwide Children's Hospital, Columbus, OH, United States; ^2^Division of Sports Medicine, Nationwide Children's Hospital, Columbus, OH, United States; ^3^Department of Pediatrics, The Ohio State University College of Medicine, Columbus, OH, United States; ^4^Center for Injury Research and Policy, Abigail Wexner Research Institute at Nationwide Children's Hospital, Columbus, OH, United States; ^5^Discipline of Children's Studies, School of Education, National University of Ireland, Galway, Ireland; ^6^Biostatistics Resource Core at Nationwide Children's Hospital, Columbus, OH, United States; ^7^Department of Psychology, Alberta Children's Hospital Research Institute, and Hotchkiss Brain Institute, University of Calgary, Calgary, AB, Canada; ^8^Department of Biostatistics, University of Nebraska Medical Center, Omaha, NE, United States; ^9^Biobehavioral Health Center, The Abigail Wexner Research Institute at Nationwide Children's Hospital, Columbus, OH, United States; ^10^Department of Epidemiology, University of Washington, Seattle, WA, United States; ^11^Division of Emergency Medicine, Nationwide Children's Hospital, Columbus, OH, United States; ^12^Department of Neurosurgery, The Ohio State University College of Medicine, Columbus, OH, United States

**Keywords:** concussion, miRNA expression, children, persistent post-concussive symptoms, saliva, biomarkers

## Abstract

**Background:**

Up to one-third of concussed children develop persistent post-concussive symptoms (PPCS). The identification of biomarkers such as salivary miRNAs that detect concussed children at increased risk of PPCS has received growing attention in recent years. However, whether and how salivary miRNA expression levels differ over time between concussed children with and without PPCS is unknown.

**Aim:**

To identify salivary MicroRNAs (miRNAs) whose expression levels differ over time post-concussion in children with vs. without PPCS.

**Methods:**

We conducted a prospective cohort study with saliva collection at up to three timepoints: (1) within one week of injury; (2) one to two weeks post-injury; and (3) 4-weeks post-injury. Participants were children (ages 11 to 17 years) with a physician-diagnosed concussion from a single hospital center. We collected participants' daily post-concussion symptom ratings throughout their enrollment using the Post-concussion Symptom Scale, and defined PPCS as a total symptom score of ≥ 5 at 28 days post-concussion. We extracted salivary RNA from the saliva samples and measured expression levels of 827 salivary miRNAs. We then compared the longitudinal expression levels of salivary miRNAs in children with vs. without PPCS using linear models with repeated measures.

**Results:**

A total of 135 saliva samples were collected from 60 children. Of the 827 miRNAs analyzed, 91 had expression levels above the calculated background threshold and were included in the differential gene expression analyses. Of these 91 miRNAs, 13 had expression levels that differed significantly across the three timepoints post-concussion between children with and without PPCS (i.e., hsa-miR-95-3p, hsa-miR-301a-5p, hsa-miR-626, hsa-miR-548y, hsa-miR-203a-5p, hsa-miR-548e-5p, hsa-miR-585-3p, hsa-miR-378h, hsa-miR-1323, hsa-miR-183-5p, hsa-miR-200a-3p, hsa-miR-888-5p, hsa-miR-199a-3p+hsa-miR-199b-3p). Among these 13 miRNAs, one (i.e., hsa-miR-203a-5p) was also identified in a prior study, with significantly different expression levels between children with and without PPCS.

**Conclusion:**

Our results from the longitudinal assessment of miRNAs indicate that the expression levels of 13 salivary miRNAs differ over time post-injury in concussed children with vs. without PPCS. Salivary miRNAs may be a promising biomarker for PPCS in children, although replication studies are needed.

## Introduction

Concussion affects approximately two million children in the United States each year (1–3). Although most concussion symptoms resolve within one to three weeks, up to one-third of children with a concussion develop persistent post-concussive symptoms (PPCS), which can last months following injury (3–5). Children with PPCS are at increased risk of experiencing missed school days, depressed mood, loss of social activities, and lower quality of life compared to children without PPCS (6–8).

Predicting clinical recovery from concussion, including identifying patients at increased risk for PPCS, is challenging (9). Several potential risk factors for PPCS in children have been identified with varying strength of association, including time from injury to initial clinical visit, acute symptom severity, vision and vestibular system dysfunction, and a history of co-morbidities such as ADHD (9–12). Currently, predicting clinical recovery from concussion is largely dependent on patient-reported symptoms and clinician-elicited signs, with the most consistent predictor of prolonged recovery being the severity of a patient's acute/subacute symptoms (10). Although increased efforts have been made to determine causes and predictors of PPCS in children, objective biomarkers that identify children at increased risk for PPCS are lacking.

MicroRNAs (miRNAs), small non-coding RNA molecules that regulate gene expression, are emerging as promising biomarkers to monitor disease, aid treatment decisions, and stratify risk, including for children with traumatic brain injury (TBI) (13–20). Prior studies show that miRNAs may control cellular processes essential to neuronal injury and repair in both the primary and secondary pathophysiology involved in TBI (21–24). Furthermore, studies have demonstrated the feasibility of identifying changes in miRNA expression associated with brain injury in biofluids, including blood and saliva (19–21, 25, 26).

Recent investigations, though mostly focused on adult patients, have shown the utility of salivary miRNA expression levels as diagnostic or prognostic biomarkers for TBI (17–19, 21, 27). In a study that included patients aged 7–21 years, Johnson et al. demonstrated overexpression of five human salivary miRNAs associated with PPCS at four weeks post-injury (18). Fedorchak et al. found an algorithm of 16 non-coding RNAs, obtained within 14 days of injury among patients aged 8–24 years, predicted concussion symptoms that lasted more than 21 days with greater accuracy than computerized balance and cognitive test performance (19). Of note, both studies included patients over the age of 18. Whether and how miRNA expression levels change over time post-injury among children aged younger than 18 years with and without PPCS remains unknown. Further studies of pediatric populations are required to confirm whether salivary miRNAs can provide accurate, objective, easily obtainable, and non-invasive biomarkers for the screening and detection of PPCS risk in children during the acute phase post-concussion.

This study aimed to longitudinally measure the expression of a panel of human salivary miRNAs at up to three timepoints post-concussion (i.e., within one week of injury, one to two weeks post-injury, and four weeks post-injury) in children aged 11–17 years, and to identify salivary miRNAs whose expression levels differ over time post-concussion in children with vs. without PPCS. The findings of this study could reveal potential biomarkers that can help detect children who may be at greater risk for PPCS in the acute or post-acute phase post-concussion. Such biomarkers could inform early, individualized, and multifaceted concussion care for children following a concussive injury (7, 19).

## Methods

### Study Design

This study involved a prospective cohort design with repeated saliva sample collection. We enrolled concussed children within 2 weeks of injury from the Emergency Department (ED) or hospital-based concussion clinics affiliated with a single children's hospital located in central Ohio (United States) and followed them until four weeks post-injury. We collected saliva samples from each participant at up to three timepoints: 1) within one week of injury (if enrollment occurred <7 days post-injury), 2) one to two weeks post-injury, and 3) four weeks post-injury. Participants also rated their daily post-concussive symptoms throughout their participation (28). We collected study data between February 2019 and November 2019. The study received ethical approval from the Institutional Review Board at the authors' hospital (IRB 18–00088). This report follows STROBE **(**STrengthening the Reporting of OBservational studies in Epidemiology) reporting guidelines for observational studies.

### Study Participants

Study participants were children aged 11 to 17 years with a physician-confirmed concussion diagnosis. Concussion was defined as a mild TBI induced by a direct or indirect blow to the head, neck, face, or other part of the body, resulting in transient neurological deficits (28). We excluded children if their concussion met any of the following criteria: 1) required surgery, 2) had any associated injury that independently interfered with neuropsychological testing (e.g., injury that affected vision), 3) was intentionally caused (i.e., resulted from assault, abuse, or self-harm), and/or 4) was associated with illicit drug or alcohol use. We also excluded children who had periodontal disease, pre-existing inflammatory/autoimmune disease(s), a current infection, or were receiving steroids/immunosuppressants; these children were excluded due to the procedures employed to collect the saliva samples (29–31). We approached 76 eligible concussed children. Of these, 66 consented. We included 60 participating children in the final analysis, excluding two participants who were lost to follow-up, and four participants who withdrew from the study due to their busy schedules.

### Study Variables and Measures

#### miRNA Expression Level

Normalized expression counts for all miRNAs on our panel were generated for each sample. The data from our saliva samples populated a database of salivary miRNA expression levels for downstream statistical evaluation and generation of a PPCS “signature” of miRNA expression. Expression levels of each of the 827 human salivary miRNAs were measured as a digital count and included in the statistical analyses as a continuous variable. Of the 827 miRNAs analyzed, 91 had expression levels above the calculated background threshold (as described in “miRNA expression assay” methods section) and were included in the results.

*Acute signs and symptoms of concussion and acute mental status* were assessed using the Standardized Assessment of Concussion (SAC), which was completed as part of routine clinical care (32). The SAC, a validated tool, includes measures of orientation, immediate memory, concentration, and delayed recall, summing to a total composite score of up to 30 points (32).

*Post-concussive symptoms* were assessed daily using the self-reported Post-concussion Symptom Scale, which is contained in the Sport Concussion Assessment Tool, 5th edition (28). The Post-concussion Symptom Scale is a validated Likert scale that measures 22 current concussion symptoms rated from 0 = no symptoms to 6 = severe symptoms. The Post-concussion Symptom Scale has established reliability (α = 0.93), construct validity, and normative data (33). Similar to prior published studies (18), we defined PPCS as a total symptom score of ≥5 at 28 days post-concussion.

*Demographic and injury variables* included age, sex, race, whether the injury occurred during a sporting event, history of prior concussion, symptom score at injury, and date of symptom resolution.

### Data Collection

Once a concussion diagnosis was confirmed, trained clinical research coordinators (at the ED) or physicians (at the concussion clinics) communicated study information to potentially eligible participants and referred them to our research team. We then contacted the child and their parent/legal guardian (“parent”) to confirm their interest and eligibility for the study and to schedule the first in-person assessment. After obtaining written informed consent/assent from a parent and the concussed child, respectively, we then collected unstimulated, non-fasting saliva using Oragene RNA RE-100 collection vials (DNA Genotek, Ottawa, ON, Canada) and completed other study assessments. We instructed participants to rinse their mouth with water prior to providing saliva and then to spit into the collection vials until the designated volume of saliva was reached (2 milliliters). We then stored the saliva samples at 4°C until they were processed for RNA isolation. We also instructed participants on how to complete online daily surveys, which were completed from the day of the initial research assessment until four weeks post-injury.

### RNA Extraction and miRNA Expression Assay

We used 2 mL of saliva to extract RNA using plasma/serum circulating and exosomal RNA slurry purification kits (Norgen Biotek, Thorold, ON, Canada). We used 300 ng of extracted RNA as input for expression of 827 different human miRNAs using the nCounter^®^ human V3 miRNA assay kit (NanoString Technologies Inc., Seattle, WA, USA), following the manufacturer's protocol. A list of all targets plus control probes can be found in Supplementary Table 1. All hybridizations were 18 h in length, and all counts were obtained by scanning on MAX mode for 555 fields of view per sample. The resulting data were analyzed using nSolver analysis software (version 4.0). We generated normalized expression counts using two parameters, positive control normalization and housekeeping normalization, as suggested by the analytical software per the manufacturer's instructions. We calculated a background “threshold” using expression values of the spike-in negative control probes (i.e., non-mammalian probes). We used the mean of the negative control counts, plus two standard deviations as our threshold. For downstream analyses, we included miRNAs only if >25% of the samples in our cohort had expression above background threshold.

### miRNA Target Gene Prediction and Functional Annotation

We used miRWalk and miRTarBase databases to identify predicted gene targets of miRNAs (34, 35). We entered each miRNA name of interest into miRWalk, an online resource that generates both predicted and validated miRNA-binding sites by identifying complementary sequence regions within known human genes. We filtered the output to include binding sites within 3'UTR and 5'UTR, and coding sequences only if they were reported as validated interactions in the miRTarBase database. The resulting gene list was used for input into The Database for Annotation, Visualization, and Integrated Discovery (DAVID; version 6.8) to assign target genes of interest to biological and functional annotations and processes (36). We considered DAVID output significant if the reported Benjamini false discovery rate (FDR) corrected *p*-value was ≤ 0.10.

### Statistical Analysis

We conducted longitudinal analyses across the three timepoints (i.e., within 1 week of injury, one to two weeks post-injury, and four weeks post-injury) to test whether miRNA expression levels differed significantly between children with and without PPCS. We first calculated the mean and standard deviation (SD) of gene expression levels of the 91 miRNAs that were above the background threshold set for our study. Next, we analyzed the longitudinal salivary miRNA expression levels using linear models with repeated measures. Due to skewed distributions of the miRNA expressions, we log-transformed miRNA expression data prior to analysis. We first modeled the expression level for each of the 91 miRNAs with main effects of time and presence of PPCS as well as the interaction between time and presence of PPCS. If the interaction term was not statistically significant, we then removed it and re-ran the longitudinal models with only the main effects of time and presence of PPCS included in the model. The FDR method was used to adjust for multiple comparisons. For each miRNA that was significantly associated with the presence of PPCS, we also tested whether their associations remained significant while adjusting for time, sex, age, history of prior concussion, and symptom score at injury by repeating similar linear models. Additionally, we assessed pair-wise interaction one at a time in each model. For models showing statistically significant interactions, we presented the results from the models including the interaction term. Finally, we characterized the miRNAs expressed in our study, and compared them to those reported in previous studies.

## Results

### Study Participants and Saliva Samples

Of the 60 participants, 32 (53.3%) were male, 55 (91.7%) were White, and 22 (36.7%) had a history of prior concussion. The mean age of participants was 14.4 (SD = 1.8) years (Table 1). Most concussions (*n* = 53, 88.3%) occurred during a sporting event, including 15 (25%) in football and 13 (21.7%) in soccer. The mean symptom score at time of injury was 46.2 (SD = 32.0). Eighteen (30.0%) participants reported persistent post-concussive symptoms at 28 days post-injury. A total of 135 saliva samples were included in our analyses. These included 29 unique samples from 29 children collected within one week of injury (27.6% of samples from children with PPCS), 47 unique samples from 47 children collected one to two weeks post-injury (29.8% of samples from children with PPCS), and 59 unique samples from 59 children collected four weeks post-injury (30.5% of samples from children with PPCS).

**Table 1:** Demographic characteristics of study participants (*N* = 60 participants).

	**Total**		**PPCS = No**		**PPCS = Yes**		P-value*
	***N*** **= 60**		***N*** **= 42**		***N*** **= 18**	
Male sex, No (%)	32	53.3	23	54.8	9	50.0	0.735
Age in years, mean (SD)	14.4	1.8	14.3	1.8	14.9	1.7	0.235
White, No (%)	54	90.0	38	90.5	16	88.9	0.850
Injured in sporting activity, No (%)	53	88.3	37	88.1	16	88.9	0.658
History of prior concussion, No (%)	20	33.3	15	35.7	5	27.8	0.550
Symptom score at injury, mean (SD)	46.2	32.0	43.0	28.3	52.5	41.2	0.331
Days from injury to enrollment, mean (SD)	7.8	3.7	7.5	3.8	8.6	3.4	0.306
Saliva samples collected at three timepoints:							
Within 1 week of injury, No (%)	29	48.3	21	72.4**	8	27.6**	
One to two weeks post-injury, No (%)	47	78.3	33	70.2**	14	29.8**	
four weeks post-injury, No (%)	59	98.3	41	69.5**	18	30.5**	

*P-values were based on chi-square tests (categorical variables) or two-sample t-tests (continuous variables).

**Row percent was presented for children with and without PPCS.

### Salivary miRNA Expression in Children With and Without PPCS

A total of 91 miRNAs were detected at expression levels above background levels and were included in the current analysis (Table 2 in the Supplementary Material). No statistically significant interactions were found between time and presence of PPCS for the 91 miRNAs, after adjusting for multiple comparisons (Table 2). Thirteen miRNAs had expression levels that differed significantly between children with and without PPCS across the three timepoints (hsa-miR-95-3p, hsa-miR-301a-5p, hsa-miR-626, hsa-miR-548y, hsa-miR-203a-5p, hsa-miR-548e-5p, hsa-miR-585-3p, hsa-miR-378h, hsa-miR-1323, hsa-miR-183-5p, hsa-miR-200a-3p, hsa-miR-888-5p, hsa-miR-199a-3p+hsa-miR-199b-3p), after adjusting for multiple comparisons (Table 2). The differences in the 13 miRNA expression levels between children with and without PPCS remained significant after adjusting for time, sex, age, history of prior concussion, and symptom score at injury (Table 2).

**Table 2:** Thirteen individual miRNAs showing significant overexpression over time post-concussion in children with persistent post-concussive symptoms (PPCS) as compared to children without PPCS, Adjusted analysis.

	**PPCS (yes vs no)**			**Timepoints**			**Sex (female vs. male)**			**Age**			**Prior concussion (yes vs. no)**			**Symptom score at injury**		
**miRNA**	**β**	**SE**	**P-value**	**β**	**SE**	**P-value**	**β**	**SE**	**P-value**	**β**	**SE**	**P-value**	**β**	**SE**	**P-value**	**β**	**SE**	**P-value**
hsa–miR−95–3p^$^	2.15	0.70	**0.00**	0.01	0.02	0.60	−0.02	0.07	0.77	0.01	0.03	0.64	−0.08	0.08	0.33	0.00	0.00	0.66
hsa–miR−301a−5p	0.69	0.15	**<0.0001**	0.03	0.02	0.13	0.11	0.14	0.45	−0.04	0.04	0.31	0.06	0.16	0.70	0.00	0.00	0.45
hsa–miR−626*	0.49	0.14	**0.00**	0.08	0.03	**0.00**	0.13	0.13	0.32	−0.01	0.04	0.69	0.29	0.18	0.11	0.00	0.00	0.82
hsa–miR−548y	0.59	0.12	**<0.0001**	0.01	0.02	0.60	0.09	0.11	0.43	−0.01	0.03	0.70	0.06	0.12	0.60	0.00	0.00	0.35
hsa–miR−203a−5p	0.62	0.13	**<0.0001**	0.03	0.02	0.25	0.11	0.12	0.35	−0.06	0.04	0.11	0.10	0.14	0.48	0.00	0.00	0.27
hsa–miR−548e−5p^$^	3.03	1.19	**0.01**	0.02	0.02	0.40	0.13	0.13	0.32	0.00	0.05	0.96	−0.03	0.14	0.86	0.00	0.00	0.58
hsa–miR−585–3p	0.59	0.14	**<0.0001**	0.03	0.02	0.22	0.13	0.13	0.30	−0.04	0.04	0.34	−0.03	0.14	0.85	0.00	0.00	0.94
hsa–miR−378h	0.41	0.13	**0.00**	0.00	0.02	0.95	0.17	0.12	0.15	0.00	0.03	0.91	−0.01	0.13	0.95	0.00	0.00	0.84
hsa–miR−1323	0.62	0.16	**0.00**	0.01	0.03	0.61	0.17	0.14	0.24	−0.04	0.04	0.32	0.05	0.16	0.73	0.00	0.00	0.63
hsa–miR−183–5p	0.65	0.14	**<0.0001**	0.02	0.03	0.38	0.03	0.13	0.84	−0.05	0.04	0.15	−0.04	0.14	0.78	0.00	0.00	0.62
hsa–miR−200a−3p^$^	2.20	0.80	**0.01**	0.00	0.02	0.78	−0.07	0.08	0.41	0.01	0.03	0.78	−0.09	0.10	0.37	0.00	0.00	0.79
hsa–miR−888–5p	0.29	0.14	**0.04**	−0.01	0.03	0.67	0.23	0.13	0.07	0.00	0.04	0.93	−0.11	0.14	0.44	0.00	0.00	0.25
hsa–miR−199a−3p+hsa–miR−199b−3p	0.68	0.14	**<0.0001**	0.01	0.02	0.59	−0.04	0.13	0.76	−0.03	0.04	0.42	0.12	0.14	0.40	0.00	0.00	0.10

*Adjusted model included the interaction between prior concussion and timepoints.

*^$^Adjusted model included the interaction between PPCS and age*.

**Figure 1:**
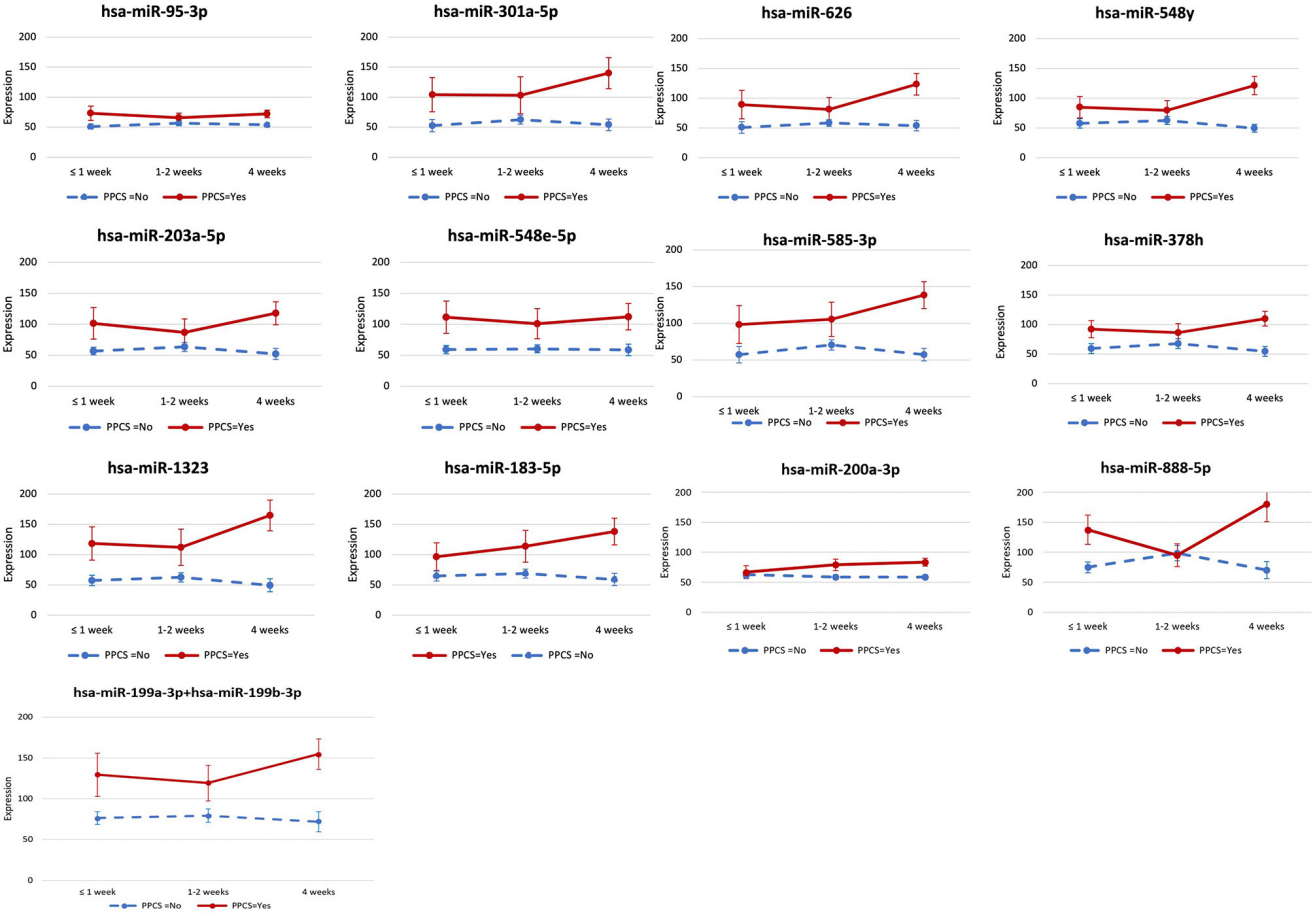
miRNA expression levels between concussed children with and without persistent post-concussive symptoms (PPCS) across the three timepoints post-injury. We identified 13 miRNAs whose expression levels were significantly higher across the three timepoints post-injury in concussed children with PPCS compared to those without PPCS. In this graph, we show the longitudinal expression levels of each miRNA at the three timepoints in concussed children with PPCS (red) vs. concussed children without PPCS (blue) without adjusting for any covariates.

The expression levels of the 13 miRNAs that were differentially expressed in the two groups were consistently higher in children with PPCS than those without PPCS across the three timepoints, except for one miRNA (hsa-miR-888-5p), which had a similar expression level in both groups at one to two weeks post-injury (Figure 1). The group differences in expression levels of the 13 miRNAs appeared to be larger at four weeks post-injury; however, the interaction between PPCS and time was not statistically significant.

**Figure 2:**
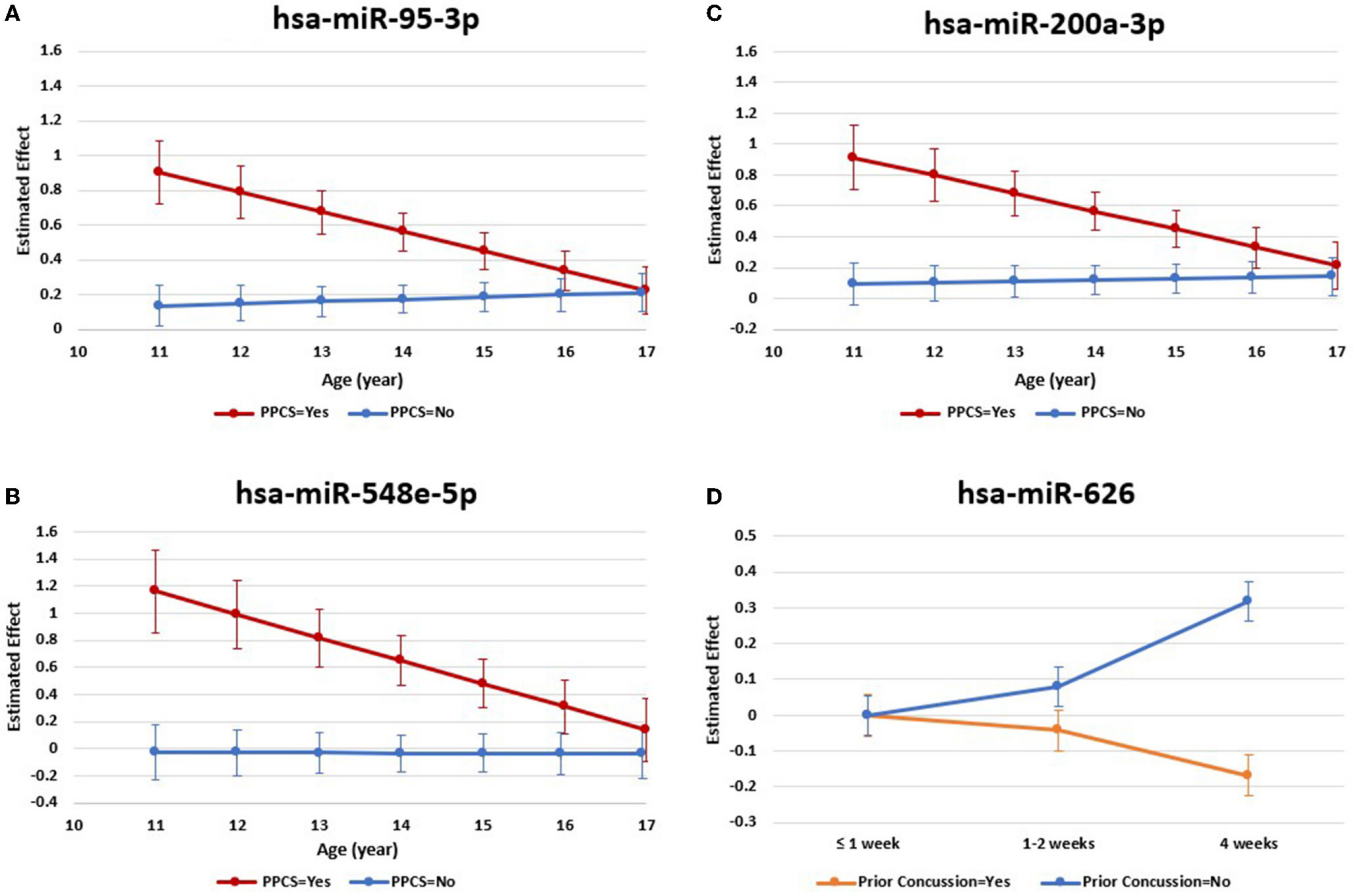
miRNA expression levels with significant interactions post-injury. We found a statistically significant interaction between age and presence of PPCS in the expression levels of three miRNAs [**(A)** hsa-miR-95-3p; **(B)** hsa-miR-548e-5p; **(C)** hsa-miR-200a-3p]. Results also revealed a statistically significant interaction between time and history of prior concussion in the expression levels of one miRNA (**D** hsa-miR-626). These results were based on adjusted models in longitudinal analyses.

We found a statistically significant interaction between age and presence of PPCS in the expression levels of three of the 13 miRNAs (hsa-miR-95-3p, hsa-miR-548e-5p, hsa-miR-200a-3p). As shown in Figures 2A–C, the relationships between expression levels of these three miRNAs and presence of PPCS differed by age. Specifically, as age increased, the expression levels of the miRNAs decreased among children with PPCS while the expression levels remained relatively stable among children without PPCS. We also found a statistically significant interaction between time and history of prior concussion for one of the 13 miRNAs (hsa-miR-626) (Figure 2D), with expression levels decreasing across timepoints in children with a history of prior concussion but increasing across timepoints in children without a history of prior concussion.

### miRNA Target Gene Prediction and Biological Annotation

**Figure 3:**
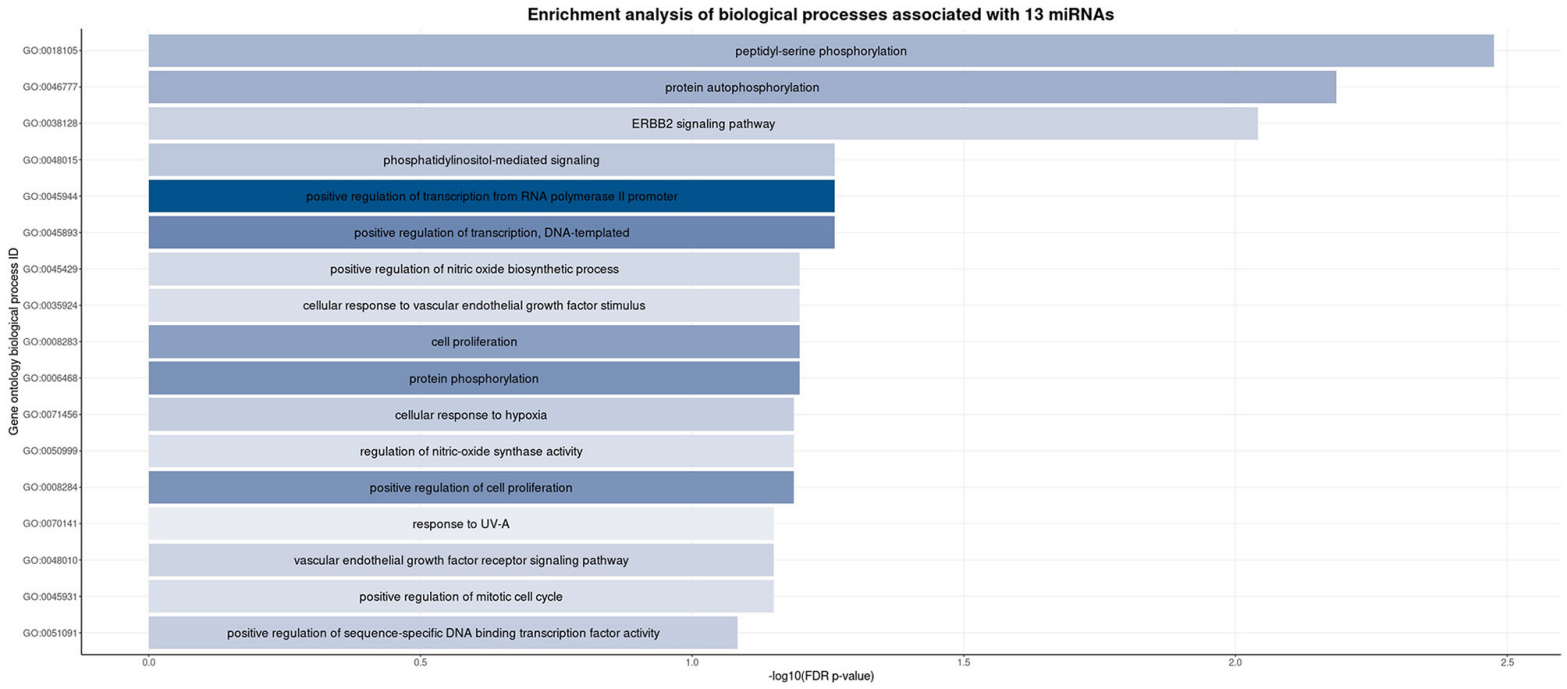
Enrichment analysis of the biological processes associated with our miRNA gene target list at three different timepoints post-injury. We used the DAVID database to upload a list of target genes based on the miRNAs found to be significantly associated with PPCS in our cohort. This figure presents the biological processes found to be significantly associated (false discovery rate *p*-value < 0.10). Each bar represents a biological process ID, whose gene ontology ID and corresponding description are listed. The width of each bar represents the –log10(FDR *p*-value) while the color of each bar indicates the count (number) of our target genes associated with that particular biological process.

The number of validated, unique target genes for all miRNAs of interest ranged from 1 to 144 (Table 3 in the Supplementary Material). We uploaded the aggregated list of miRNA target genes to the DAVID functional annotation webtool to identify any biological process(es) for which the gene list was enriched (Figure 3). These results indicated that many of the target genes are involved in phosphorylation, transcriptional regulation, and cell signaling pathways.

### miRNA Expressed in the Current Study as Compared to Prior Published Studies

Three miRNAs found to be associated with PPCS in prior studies were available in our dataset (after quality control) for replication (i.e., hsa-miR-203a-5p, hsa-miR-148-3p, hsa-miR-1246) (Table 3). Of these three miRNAs, one (i.e., has-miR-203a-5p) showed significant association with PPCS in the current study and a prior study.^19^

**Table 3:** Previously identified potential salivary miRNA biomarkers associated with persistent post–concussive symptoms (PPCS).

	**Association in other studies**	**Associated with PPCS in our cohort?**	**P–value in current study**
**Previously identified salivary miRNA that are expressed in current study**			
hsa–miR−203a−5p	PPCS (19)	Yes	**0.00319**
hsa–miR−148–3p	PPCS (19)	No	hsa–miR−148a−3p: 0.28106 hsa–miR−148b−3p: 0.52139
hsa–miR−1246	PPCS (19)	No	0.85564
**Previously identified salivary miRNA that are not expressed or not measured in current study**			
hsa–miR−320c−1	PPCS (18)	No	NA
hsa–miR−133a−5p	PPCS (18)	No	NA
hsa–miR−769–5p	PPCS (18)	No	NA
hsa–let−7a−3p	PPCS (18)	Not measured	NA
hsa–miR−100–5p	PPCS (19)	No	NA
hsa–miR−148a−5p	PPCS (19)	Not measured	NA
hsa–miR−423–5p	PPCS (19)	No	NA
hsa–miR−92b−3p	PPCS (19)	No	NA
hsa–miR−1307–3p	PPCS (18)	No	NA
**Salivary miRNA significantly expressed in current study that were not previously identified**			
hsa–miR−548y	NA	Yes	**0.00015**
hsa–miR−585–3p	NA	Yes	**0.00015**
hsa–miR−378h	NA	Yes	**0.00015**
hsa–miR−1323	NA	Yes	**0.00015**
hsa–miR−183–5p	NA	Yes	**0.00015**
hsa–miR−199a−3p+hsa–miR−199b−3p	NA	Yes	**0.00015**
hsa–miR−301a−5p	NA	Yes	**0.00148**
hsa–miR−626	NA	Yes	**0.00244**
hsa–miR−888–5p	NA	Yes	**0.00518**
hsa–miR−548e−5p	NA	Yes	**0.00696**
hsa–miR−200a−3p	NA	Yes	**0.00789**
hsa–miR−95–3p	NA	Yes	**0.01010**

## Discussion

This study, to the best of our knowledge, is the first to longitudinally assess a panel of human salivary miRNA expression levels over time in a sample consisting exclusively of children after concussion (37). Of the 91 miRNAs expressed above background levels, 13 were significantly upregulated post-concussion in children with PPCS as compared to those without PPCS. Expression levels of these 13 miRNAs were higher in children with PPCS than those without PPCS across the three timepoints. Our findings add to a growing body of knowledge regarding changes in salivary miRNA expression levels among children post-concussion (17–19, 21, 22). These findings also add to previous work demonstrating the potential for miRNAs to discriminate between concussed children with and without PPCS (19). Identifying potential objective prognostic biomarkers of PPCS may help clinicians detect children at greater risk of PPCS during the acute post-injury phase (18, 19), and thereby facilitate early, individualized treatment plans for concussed children (7–12). The non-invasive nature of sampling for salivary miRNA makes this especially appealing when caring for children.

Three of the 91 miRNAs expressed in a prior study of PPCS by Fedorchak et al. (19) were also expressed in the current study. However, only one (hsa-miR-203a-5p) of these three miRNAs demonstrated significant overexpression across the three timepoints post-concussion in children with PPCS. These findings reflect the inconsistency among previous studies regarding miRNA expression levels such that salivary miRNAs may be overexpressed in children with PPCS in one study but not in other studies (18, 19). Such inconsistencies in study findings may be due, in part, to differences in the study populations, sample size, number and time of data collection, definition of PPCS, or analytic approaches employed. For example, Johnson et al. (18) only collected one salivary sample per participant, while Fedorchak's (19) and our study collected multiple salivary samples from each participant. Further, the definition of PPCS used in Johnson's (18) and our study (i.e., symptom presentation at ≥28 days post-injury) differed from the definition used by Fedorchak et al. (19) (i.e., symptom presentation at ≥ 21 days post-injury. In addition, it is important to consider matching the age of patients in future studies as we found a statistically significant interaction between age and presence of PPCS in the expression levels of three miRNAs. While there are currently no databases which catalog salivary miRNA expression levels in pediatric patients without disease, there is evidence of age-related effects on expression levels of miRNA in both saliva (38, 39) and serum (40) samples. As research on biomarkers for the prognosis of concussion is still in its infancy, further verification of these findings in a larger, more diverse cohort of concussed children and with standardized data collection tools and uniform analytic approaches is needed. Further, since biological changes do not provide information about the clinical manifestation of concussion (7, 8, 13), future studies should validate our findings with established, standardized clinical measures (e.g., balance and cognitive testing) or risk for PPCS (e.g., 5P Risk Score) to ensure the clinical utility of these biomarkers (7, 19). Our findings, along with others (13–19, 41), highlight the need for more research, with larger samples, to determine whether salivary miRNAs can accurately predict and detect PPCS in concussed children. If confirmed in other studies, these findings could inform the design of clinical assays (e.g., saliva collection) for use as an objective, non-invasive, and easily administrated test for children with concussion.

This study contributes to scientific knowledge on miRNA target gene prediction and biological annotation. The miRNAs that accurately discriminated between children with and without PPCS over time post-injury target genes that are significantly enriched for many biological processes, including protein phosphorylation, cell signaling pathways, and transcriptional regulation. The different distributions of the identified salivary miRNAs in children with vs. without PPCS may signal a differential physiological response to the concussive injury or its subsequent repair. However, more research is needed to better understand the functional effects of miRNAs on their target genes, specifically within the context of concussion.

We found no statistically significant associations between symptom score at injury and the expression levels of the 13 miRNAs that were differentially expressed in concussed children with vs. without PPCS. This result may suggest that the 13 miRNAs that were overexpressed in the current study may serve as prognostic biomarkers for PPCS independent of acute symptom severity (13, 17–19, 26). Additional studies with more participants and multiple timepoints are needed to confidently identify all miRNAs that demonstrate an altered expression level following concussion as well as those associated with an increased risk for PPCS. Moreover, future studies are needed to further our understanding of the biological mechanisms of salivary miRNAs in children with concussion.

This study is not without limitations. First, the study had a relatively small sample size, which was relatively lacking in diversity, consisting almost exclusively of White participants from a single hospital center. Second, saliva samples were not available from all participants at all three timepoints, although our analytic approach accounted for this limitation. Third, we did not have knowledge about the resting expression levels of salivary miRNAs in age-matched children without concussion. Fourth, regarding functional annotation, while several databases curate both validated and predicted binding sites of miRNAs, these databases are not comprehensive, and no single centralized database houses all known cataloged gene targets (32, 33, 42–45). This is an evolving field, as we have noted. Fifth, while we restricted our target gene search to only those reported as validated by *in vitro* assays, some miRNAs are better studied than others, especially if they are implicated in more commonly studied diseases like cancer. Finally, participants were from hospital-based ED and concussion clinics, which potentially may treat more severe concussive injuries; thus, our findings may not readily generalize to concussed children seen in primary care clinics or other clinical settings, nor to children who are 10 years of age or younger. Despite these limitations, our study contributes to an emerging body of knowledge on potential biomarkers for the prognosis of pediatric concussion by longitudinally assessing miRNA expression levels over time post-concussion in children aged 11–17 years old and by exploring the physiological processes of the significantly upregulated miRNAs.

In conclusion, this study demonstrated that 13 salivary miRNAs were significantly upregulated post-concussion in children with PPCS as compared to their counterparts, suggesting that salivary miRNAs may serve as an objective prognostic biomarker for PPCS. Additional research is needed to determine the ability of salivary miRNA biomarkers to enhance the early prediction of PPCS risk in concussed children, including studies that verify our findings in larger, more diverse cohorts and with standardized clinical assessment tools. Although research on biomarkers for the prognosis of concussion is still in its infancy, salivary biomarkers hold promise for assisting in the early identification and treatment of children at increased risk for PPCS.

## Data Availability Statement

The original contributions presented in the study are included in the article/Supplementary Material, further inquiries can be directed to the corresponding author.

## Ethics Statement

The studies involving human participants were reviewed and approved by Institutional Review Board at Abigail Wexner Research Institute at Nationwide Children's Hospital. Written informed consent to participate in this study was provided by the participants' legal guardian/next of kin.

## NCH Concussion Research Group

Steven C. Cuff, MD; Drew Duerson, MD; Anastasia N. Fischer, MD; Jonathan T. Napolitano, MD; Reno Ravindran, MD; Richard E. Rodenberg Jr., MD; Amy E. Valasek, MD; Division of Sports Medicine, Nationwide Children's Hospital and Department of Pediatrics, The Ohio State University, College of Medicine, Columbus, Ohio, USA

## Author Contributions

JY, EM, KM, and LS contributed to concept and design. JM, LS, AH, NA, DC, and TP collected study data. KM, LV, JY, LS, KY, HT, SC, JM, and EM contributed to analysis and interpretation of data. SC, EA, and JS conducted statistical analysis. KM, JY, LS, and JM drafted the initial manuscript. All authors contributed to critical revision of the manuscript for important intellectual content and approved the final manuscript as submitted and agree to be accountable for all aspects of the work.

## Funding

Research reported in this publication was supported by Nationwide Children's Hospital's Intramural Funding Program and by The Ohio State University's Discovery Theme Initiative in Chronic Brain Injury. The funders had no role in the design and conduct of the study.

## Author Disclaimer

The content is solely the responsibility of the authors and does not necessarily represent the official views of the funders.

## Conflict of Interest

The authors declare that the research was conducted in the absence of any commercial or financial relationships that could be construed as a potential conflict of interest. The reviewer RK declared a shared affiliation with co-author KY to the handling editor at the time of review.

## Publisher's Note

All claims expressed in this article are solely those of the authors and do not necessarily represent those of their affiliated organizations, or those of the publisher, the editors and the reviewers. Any product that may be evaluated in this article, or claim that may be made by its manufacturer, is not guaranteed or endorsed by the publisher.

## Abbreviations

ED: emergency department
FDR: false discovery rate
miRNA: microRNA
PPCS: persistent post-concussive symptoms
SD: standard deviation
TBI: traumatic brain injury.
