# Supplementary material for: Salivary miRNA Expression in Children With Persistent Post-concussive Symptoms
Source: Front Public Health. 2022 May 30;10:890420. doi: 10.3389/fpubh.2022.890420 (PMC9195510; doi:10.3389/fpubh.2022.890420)
Supplement: Supplementary file 1 [file Table_1.pdf]

eTable 1. List of targets in the NanoString human v3 miRNA expression assay

Official symbol	Accession number	Target Sequence
hsa-let-7a-5p	MIMAT0000062	UGAGGUAGUAGGUUGUAUAGUU
hsa-let-7b-5p	MIMAT0000063	UGAGGUAGUAGGUUGUGUGGUU
hsa-let-7c-5p	MIMAT0000064	UGAGGUAGUAGGUUGUAUGGUU
hsa-let-7d-5p	MIMAT0000065	AGAGGUAGUAGGUUGCAUAGUU
hsa-let-7e-5p	MIMAT0000066	UGAGGUAGGAGGUUGUAUAGUU
hsa-let-7f-5p	MIMAT0000067	UGAGGUAGUAGAUUGUAUAGUU
hsa-miR-15a-5p	MIMAT0000068	UAGCAGCACAUAAUGGUUUGUG
hsa-miR-16-5p	MIMAT0000069	UAGCAGCACGUAAAUAUUGGCG
hsa-miR-17-5p	MIMAT0000070	CAAAGUGCUUACAGUGCAGGUAG
hsa-miR-18a-5p	MIMAT0000072	UAAGGUGCAUCUAGUGCAGAUAG
hsa-miR-19a-3p	MIMAT0000073	UGUGCAAAUCUAUGCAAAACUGA
hsa-miR-19b-3p	MIMAT0000074	UGUGCAAAUCCAUGCAAAACUGA
hsa-miR-20a-5p	MIMAT0000075	UAAAGUGCUUAUAGUGCAGGUAG
hsa-miR-21-5p	MIMAT0000076	UAGCUUAUCAGACUGAUGUUGA
hsa-miR-22-3p	MIMAT0000077	AAGCUGCCAGUUGAAGAACUGU
hsa-miR-23a-3p	MIMAT0000078	AUCACAUUGCCAGGGAUUUCC
hsa-miR-24-3p	MIMAT0000080	UGGCUCAGUUCAGCAGGAACAG
hsa-miR-25-3p	MIMAT0000081	CAUUGCACUUGUCUCGGUCUGA
hsa-miR-26a-5p	MIMAT0000082	UUCAAGUAAUCCAGGAUAGGCU
hsa-miR-26b-5p	MIMAT0000083	UUCAAGUAAUUCAGGAUAGGU
hsa-miR-27a-3p	MIMAT0000084	UUCACAGUGGCUAAGUUCCGC
hsa-miR-28-5p	MIMAT0000085	AAGGAGCUCACAGUCUAUUGAG
hsa-miR-29a-3p	MIMAT0000086	UAGCACCAUCUGAAAUCGGUUA
hsa-miR-30a-5p	MIMAT0000087	UGUAAACAUCCUCGACUGGAAG
hsa-miR-30a-3p	MIMAT0000088	CUUUCAGUCGGAUGUUUGCAGC
hsa-miR-31-5p	MIMAT0000089	AGGCAAGAUGCUGGCAUAGCU
hsa-miR-32-5p	MIMAT0000090	UAUUGCACAUUACUAAGUUGCA
hsa-miR-33a-5p	MIMAT0000091	GUGCAUUGUAGUUGCAUUGCA
hsa-miR-92a-3p	MIMAT0000092	UAUUGCACUUGUCCCGGCCUGU
hsa-miR-93-5p	MIMAT0000093	CAAAGUGCUGUUCGUGCAGGUAG
hsa-miR-95-3p	MIMAT0000094	UUCAACGGGUAUUUAUUGAGCA
hsa-miR-96-5p	MIMAT0000095	UUUGGCACUAGCACAUUUUUGCU
hsa-miR-98-5p	MIMAT0000096	UGAGGUAGUAAGUUGUAUUGUU
hsa-miR-99a-5p	MIMAT0000097	AACCCGUAGAUCCGAUCUUGUG
hsa-miR-100-5p	MIMAT0000098	AACCCGUAGAUCCGAACUUGUG
hsa-miR-101-3p	MIMAT0000099	UACAGUACUGUGAUAACUGAA

Official symbol	Accession number	Target Sequence
hsa-miR-29b-3p	MIMAT0000100	UAGCACCAUUUGAAAUCAGUGUU
hsa-miR-103a-3p	MIMAT0000101	AGCAGCAUUGUACAGGGCUAUGA
hsa-miR-105-5p	MIMAT0000102	UCAAAUGCUCAGACUCCUGUGGU
hsa-miR-106a-5p	MIMAT0000103	AAAAGUGCUUACAGUGCAGGUAG
hsa-miR-107	MIMAT0000104	AGCAGCAUUGUACAGGGCUAUCA
hsa-miR-192-5p	MIMAT0000222	CUGACCUAUGAAUUGACAGCC
hsa-miR-196a-5p	MIMAT0000226	UAGGUAGUUUCAUGUUGUUGGG
hsa-miR-197-3p	MIMAT0000227	UUCACCACCUUCUCCACCCAGC
hsa-miR-198	MIMAT0000228	GGUCCAGAGGGGAGAUAGGUUC
hsa-miR-199a-5p	MIMAT0000231	CCCAGUGUUCAGACUACCUGUUC
hsa-miR-199a-3p	MIMAT0000232	ACAGUAGUCUGCACAUUGGUUA
hsa-miR-208a-3p	MIMAT0000241	AUAAGACGAGCAAAAAGCUUGU
hsa-miR-129-5p	MIMAT0000242	CUUUUUGCGGUCUGGGCUUGC
hsa-miR-148a-3p	MIMAT0000243	UCAGUGCACUACAGAACUUUGU
hsa-miR-30c-5p	MIMAT0000244	UGUAAACAUCCUACACUCUCAGC
hsa-miR-30d-5p	MIMAT0000245	UGUAAACAUCCCCGACUGGAAG
hsa-miR-139-5p	MIMAT0000250	UCUACAGUGCACGUGUCUCCAG
hsa-miR-147a	MIMAT0000251	GUGUGUGGAAAUGCUUCUGC
hsa-miR-7-5p	MIMAT0000252	UGGAAGACUAGUGAUUUUGUUGU
hsa-miR-10a-5p	MIMAT0000253	UACCCUGUAGAUCCGAAUUUGUG
hsa-miR-10b-5p	MIMAT0000254	UACCCUGUAGAACCGAAUUUGUG
hsa-miR-34a-5p	MIMAT0000255	UGGCAGUGUCUUAGCUGGUUGU
hsa-miR-181a-5p	MIMAT0000256	AACAUUCAACGCUGUCGGUGAGU
hsa-miR-181b-5p	MIMAT0000257	AACAUUCAUUGCUGUCGGUGGGU
hsa-miR-181c-5p	MIMAT0000258	AACAUUCAACCUGUCGGUGAGU
hsa-miR-182-5p	MIMAT0000259	UUUGGCAAUGGUAGAACUCACACU
hsa-miR-182-3p	MIMAT0000260	UGGUUCUAGACUUGCCAACUA
hsa-miR-183-5p	MIMAT0000261	UAUGGCACUGGUAGAAUUCACU
hsa-miR-187-3p	MIMAT0000262	UCGUGUCUUGUGUUGCAGCCGG
hsa-miR-199b-5p	MIMAT0000263	CCCAGUGUUUAGACUAUCUGUUC
hsa-miR-203a-3p	MIMAT0000264	GUGAAAUGUUUAGGACCACUAG
hsa-miR-204-5p	MIMAT0000265	UUCCCUUUGUCAUCCUAUGCCU
hsa-miR-205-5p	MIMAT0000266	UCCUUCAUUCCACCGGAGUCUG
hsa-miR-210-3p	MIMAT0000267	CUGUGCGUGUGACAGCGGCUGA
hsa-miR-211-5p	MIMAT0000268	UUCCCUUUGUCAUCCUUCGCCU
hsa-miR-212-3p	MIMAT0000269	UAACAGUCUCCAGUCACGGCC
hsa-miR-181a-3p	MIMAT0000270	ACCAUCGACCGUUGAUUGUACC

Official symbol	Accession number	Target Sequence
hsa-miR-214-3p	MIMAT0000271	ACAGCAGGCACAGACAGGCAGU
hsa-miR-215-5p	MIMAT0000272	AUGACCUAUGAAUUGACAGAC
hsa-miR-216a-5p	MIMAT0000273	UAAUCUCAGCUGGCAACUGUGA
hsa-miR-217	MIMAT0000274	UACUGCAUCAGGAACUGAUUGGA
hsa-miR-218-5p	MIMAT0000275	UUGUGCUUGAUCUAACCAUGU
hsa-miR-219a-5p	MIMAT0000276	UGAUUGUCCAAACGCAAUUCU
hsa-miR-221-3p	MIMAT0000278	AGCUACAUUGUCUGCUGGGUUUC
hsa-miR-222-3p	MIMAT0000279	AGCUACAUCUGGCUACUGGGU
hsa-miR-223-3p	MIMAT0000280	UGUCAGUUUGUCAAAUACCCCA
hsa-miR-224-5p	MIMAT0000281	CAAGUCACUAGUGGUUCCGUU
hsa-miR-200b-3p	MIMAT0000318	UAAUACUGCCUGGUAAUGAUGA
hsa-let-7g-5p	MIMAT0000414	UGAGGUAGUAGUUUGUACAGUU
hsa-let-7i-5p	MIMAT0000415	UGAGGUAGUAGUUUGUGCUGUU
hsa-miR-1-3p	MIMAT0000416	UGGAAUGUAAAGAAGUAUGUAU
hsa-miR-15b-5p	MIMAT0000417	UAGCAGCACAUCAUGGUUUACA
hsa-miR-23b-3p	MIMAT0000418	AUCACAUUGCCAGGGAUUACC
hsa-miR-27b-3p	MIMAT0000419	UUCACAGUGGCUAAGUUCUGC
hsa-miR-30b-5p	MIMAT0000420	UGUAAACAUCCUACACUCAGCU
hsa-miR-122-5p	MIMAT0000421	UGGAGUGUGACAAUGGUGUUUG
hsa-miR-124-3p	MIMAT0000422	UAAGGCACGCGGUGAAUGCC
hsa-miR-125b-5p	MIMAT0000423	UCCCUGAGACCCUAACUUGUGA
hsa-miR-128-3p	MIMAT0000424	UCACAGUGAACCGGUCUCUUU
hsa-miR-130a-3p	MIMAT0000425	CAGUGCAAUGUUAAAAGGGCAU
hsa-miR-132-3p	MIMAT0000426	UAACAGUCUACAGCCAUGGUCG
hsa-miR-133a-3p	MIMAT0000427	UUUGGUCCCCUUCAACCAGCUG
hsa-miR-135a-5p	MIMAT0000428	UAUGGCUUUUUAUUCCUAUGUGA
hsa-miR-137	MIMAT0000429	UUAUUGCUUAAGAAUACGCGUAG
hsa-miR-138-5p	MIMAT0000430	AGCUGGUGUUGUGAAUCAGGCCG
hsa-miR-140-5p	MIMAT0000431	CAGUGGUUUUACCCUAUGGUAG
hsa-miR-141-3p	MIMAT0000432	UAACACUGUCUGGUAAAGAUGG
hsa-miR-142-5p	MIMAT0000433	CAUAAAGUAGAAAGCACUACU
hsa-miR-142-3p	MIMAT0000434	UGUAGUGUUUCCUACUUUAUGGA
hsa-miR-143-3p	MIMAT0000435	UGAGAUGAAGCACUGUAGCUC
hsa-miR-144-3p	MIMAT0000436	UACAGUAUAGAUGAUGUACU
hsa-miR-145-5p	MIMAT0000437	GUCCAGUUUUCCCAGGAAUCCCU
hsa-miR-152-3p	MIMAT0000438	UCAGUGCAUGACAGAACUUGG
hsa-miR-153-3p	MIMAT0000439	UUGCAUAGUCACAAAAGUGAUC

Official symbol	Accession number	Target Sequence
hsa-miR-191-5p	MIMAT0000440	CAACGGAAUCCCAAAAGCAGCUG
hsa-miR-9-5p	MIMAT0000441	UCUUUGGUUAUCUAGCUGUAUGA
hsa-miR-125a-5p	MIMAT0000443	UCCCUGAGACCCUUUAACCUGUGA
hsa-miR-126-3p	MIMAT0000445	UCGUACCGUGAGUAAUAAUGCG
hsa-miR-127-3p	MIMAT0000446	UCGGAUCCGUCUGAGCUUGGCU
hsa-miR-134-5p	MIMAT0000447	UGUGACUGGUUGACCAGAGGGG
hsa-miR-136-5p	MIMAT0000448	ACUCCAUUUGUUUUGAUGAUGGA
hsa-miR-146a-5p	MIMAT0000449	UGAGAACUGAAUUCCAUGGGUU
hsa-miR-149-5p	MIMAT0000450	UCUGGCUCCGUGUCUUCACUCCC
hsa-miR-150-5p	MIMAT0000451	UCUCCCAACCCUUGUACCAGUG
hsa-miR-154-5p	MIMAT0000452	UAGGUUAUCCGUGUUGCCUUCG
hsa-miR-184	MIMAT0000454	UGGACGGAGAACUGAUAAGGGU
hsa-miR-185-5p	MIMAT0000455	UGGAGAGAAAGGCAGUUCCUGA
hsa-miR-186-5p	MIMAT0000456	CAAAGAAUUCUCCUUUUGGGCU
hsa-miR-188-5p	MIMAT0000457	CAUCCCUUGCAUGGUGGAGGG
hsa-miR-190a-5p	MIMAT0000458	UGAUAUGUUUGAUAUAUUAGGU
hsa-miR-193a-3p	MIMAT0000459	AACUGGCCUACAAAGUCCCAGU
hsa-miR-194-5p	MIMAT0000460	UGUAACAGCAACUCCAUGUGGA
hsa-miR-195-5p	MIMAT0000461	UAGCAGCACAGAAAUAUUGGC
hsa-miR-206	MIMAT0000462	UGGAAUGUAAGGAAGUGUGUGG
hsa-miR-320a	MIMAT0000510	AAAAGCUGGGUUGAGAGGGCGA
hsa-miR-200c-3p	MIMAT0000617	UAAUACUGCCGGGUAAUGAUGGA
hsa-miR-155-5p	MIMAT0000646	UUAAUGCUAAUCGUGAUAGGGGU
hsa-miR-106b-5p	MIMAT0000680	UAAAGUGCUGACAGUGCAGAU
hsa-miR-29c-3p	MIMAT0000681	UAGCACCAUUUGAAAUCGGUUA
hsa-miR-200a-3p	MIMAT0000682	UAACACUGUCUGGUAACGAUGU
hsa-miR-302a-5p	MIMAT0000683	ACUUAAACGUGGAUGUACUUGCU
hsa-miR-302a-3p	MIMAT0000684	UAAGUGCUUCCAUGUUUUGGUGA
hsa-miR-34c-5p	MIMAT0000686	AGGCAGUGUAGUUAGCUGAUUGC
hsa-miR-299-3p	MIMAT0000687	UAUGUGGGAUGGUAAACCGCUU
hsa-miR-301a-3p	MIMAT0000688	CAGUGCAAUAGUAUUGUCAAAGC
hsa-miR-99b-5p	MIMAT0000689	CACCCGUAGAACCGACCUUGCG
hsa-miR-296-5p	MIMAT0000690	AGGGCCCCCCCUCAAUCCUGU
hsa-miR-130b-3p	MIMAT0000691	CAGUGCAAUGAUGAAAGGGCAU
hsa-miR-30e-5p	MIMAT0000692	UGUAAACAUCCUUGACUGGAAG
hsa-miR-30e-3p	MIMAT0000693	CUUUCAGUCGGAUGUUUACAGC
hsa-miR-361-5p	MIMAT0000703	UUAUCAGAAUCUCCAGGGGUAC

Official symbol	Accession number	Target Sequence
hsa-miR-362-5p	MIMAT0000705	AAUCCUUGGAACCUAGGUGUGAGU
hsa-miR-363-3p	MIMAT0000707	AAUUGCACGGUAUCCAUCUGUA
hsa-miR-365a-3p	MIMAT0000710	UAAUGCCCCUAAAAAUCCUUAU
hsa-miR-302b-3p	MIMAT0000715	UAAGUGCUUCCAUGUUUUAGUAG
hsa-miR-302c-3p	MIMAT0000717	UAAGUGCUUCCAUGUUUCAGUGG
hsa-miR-302d-3p	MIMAT0000718	UAAGUGCUUCCAUGUUUGAGUGU
hsa-miR-367-3p	MIMAT0000719	AAUUGCACUUUAGCAAUGGUGA
hsa-miR-376c-3p	MIMAT0000720	AACAUAGAGGAAAUUCCACGU
hsa-miR-369-3p	MIMAT0000721	AAUAAUACAUGGUUGAUCUUU
hsa-miR-370-3p	MIMAT0000722	GCCUGCUGGGGUGGAACCUGGU
hsa-miR-372-3p	MIMAT0000724	AAAGUGCUGCGACAUUUGAGCGU
hsa-miR-373-3p	MIMAT0000726	GAAGUGCUUCGAUUUUGGGGUGU
hsa-miR-374a-5p	MIMAT0000727	UUAUAAUACAACCUGAUAAGUG
hsa-miR-375	MIMAT0000728	UUUGUUCGUUCGGCUCGCGUGA
hsa-miR-376a-3p	MIMAT0000729	AUCAUAGAGGAAAAUCCACGU
hsa-miR-377-3p	MIMAT0000730	AUCACACAAAGGCAACUUUUGU
hsa-miR-379-5p	MIMAT0000733	UGGUAGACUAUGGAACGUAGG
hsa-miR-380-3p	MIMAT0000735	UAUGUAAUAUGGUCCACAUCUU
hsa-miR-381-3p	MIMAT0000736	UAUACAAGGGCAAGCUCUCUGU
hsa-miR-382-5p	MIMAT0000737	GAAGUUGUUCGUGGUGGAUUCG
hsa-miR-383-5p	MIMAT0000738	AGAUCAGAAGGUGAUUGUGGCU
hsa-miR-330-3p	MIMAT0000751	GCAAAGCACACGGCCUGCAGAGA
hsa-miR-328-3p	MIMAT0000752	CUGGCCCUCUCUGCCCUUCCGU
hsa-miR-342-3p	MIMAT0000753	UCUCACACAGAAAUCGCACCCGU
hsa-miR-337-3p	MIMAT0000754	CUCCUAUAUGAUGCCUUUCUUC
hsa-miR-323a-3p	MIMAT0000755	CACAUUACACGGUCGACCUCU
hsa-miR-326	MIMAT0000756	CCUCUGGGCCCUUCCUCCAG
hsa-miR-151a-3p	MIMAT0000757	CUAGACUGAAGCUCCUUGAGG
hsa-miR-135b-5p	MIMAT0000758	UAUGGCUUUUCAUUCCUAUGUGA
hsa-miR-148b-3p	MIMAT0000759	UCAGUGCAUCACAGAACUUUGU
hsa-miR-331-3p	MIMAT0000760	GCCCCUGGGCCUAUCCUAGAA
hsa-miR-324-5p	MIMAT0000761	CGCAUCCCCUAGGGCAUUGGUGU
hsa-miR-324-3p	MIMAT0000762	ACUGCCCCAGGUGCUGCUGG
hsa-miR-339-5p	MIMAT0000764	UCCCUGUCCUCCAGGAGCUCACG
hsa-miR-335-5p	MIMAT0000765	UCAAGAGCAAUAACGAAAAAUGU
hsa-miR-133b	MIMAT0000770	UUUGGUCCCCUUCAACCAGCUA
hsa-miR-325	MIMAT0000771	CCUAGUAGGUGUCCAGUAAGUGU

Official symbol	Accession number	Target Sequence
hsa-miR-345-5p	MIMAT0000772	GCUGACUCCUAGUCCAGGGCUC
hsa-miR-346	MIMAT0000773	UGUCUGCCCGCAUGCCUGCCUCU
hsa-miR-384	MIMAT0001075	AUUCCUAGAAAUUGUUCAUA
hsa-miR-196b-5p	MIMAT0001080	UAGGUAGUUUCCUGUUGUUGGG
hsa-miR-422a	MIMAT0001339	ACUGGACUUAGGGUCAGAAGGC
hsa-miR-423-3p	MIMAT0001340	AGCUCGGUCUGAGGCCCCUCAGU
hsa-miR-424-5p	MIMAT0001341	CAGCAGCAAUUCAUGUUUUGAA
hsa-miR-18b-5p	MIMAT0001412	UAAGGUGCAUCUAGUGCAGUUAG
hsa-miR-20b-5p	MIMAT0001413	CAAAGUGCUCAUAGUGCAGGUAG
hsa-miR-448	MIMAT0001532	UUGCAUAUGUAGGAUGUCCCAU
hsa-miR-429	MIMAT0001536	UAAUACUGUCUGGUAAAACCGU
hsa-miR-449a	MIMAT0001541	UGGCAGUGUAUUGUUAGCUGGU
hsa-miR-450a-5p	MIMAT0001545	UUUUGCGAUGUGUUCCUAAUAU
hsa-miR-369-5p	MIMAT0001621	AGAUCGACCGUGUUAUAUUCGC
hsa-miR-431-5p	MIMAT0001625	UGUCUUGCAGGCCGUCAUGCA
hsa-miR-433-3p	MIMAT0001627	AUCAUGAUGGGCUCCUCGGUGU
hsa-miR-329-3p	MIMAT0001629	AACACACCUGGUUAACCUCUUU
hsa-miR-323b-5p	MIMAT0001630	AGGUUGUCCGUGGUGAGUUCGCA
hsa-miR-451a	MIMAT0001631	AAACCGUUACCAUUACUGAGUU
hsa-miR-452-5p	MIMAT0001635	AACUGUUUGCAGAGGAAACUGA
hsa-miR-409-5p	MIMAT0001638	AGGUUACCCGAGCAACUUUGCAU
hsa-miR-409-3p	MIMAT0001639	GAAUGUUGCUCGGUGAACCCCU
hsa-miR-412-3p	MIMAT0002170	ACUUCACCUGGUCCACUAGCCGU
hsa-miR-410-3p	MIMAT0002171	AAUAUAACACAGAUGGCCUGU
hsa-miR-376b-3p	MIMAT0002172	AUCAUAGAGGAAAAUCCAUGUU
hsa-miR-483-3p	MIMAT0002173	UCACUCCUCUCCUCCCGUCUU
hsa-miR-484	MIMAT0002174	UCAGGCUCAGUCCCCUCCCGAU
hsa-miR-485-5p	MIMAT0002175	AGAGGCUGGCCGUGAUGAAUUC
hsa-miR-485-3p	MIMAT0002176	GUCAUACACGGCUCUCCUCUCU
hsa-miR-487a-3p	MIMAT0002178	AAUCAUACAGGGACAUCCAGUU
hsa-miR-489-3p	MIMAT0002805	GUGACAUCACAUAUACGGCAGC
hsa-miR-490-3p	MIMAT0002806	CAACCUGGAGGACUCCAUGCUG
hsa-miR-491-5p	MIMAT0002807	AGUGGGGAACCCUUCCAUGAGG
hsa-miR-511-5p	MIMAT0002808	GUGUCUUUUGCUCUGCAGUCA
hsa-miR-146b-5p	MIMAT0002809	UGAGAACUGAAUUCCAUAGGCU
hsa-miR-202-3p	MIMAT0002811	AGAGGUAUAGGGCAUGGGAA
hsa-miR-492	MIMAT0002812	AGGACCUGCGGGACAAGAUUCUU

Official symbol	Accession number	Target Sequence
hsa-miR-432-5p	MIMAT0002814	UCUUGGAGUAGGUCAUUGGGUGG
hsa-miR-494-3p	MIMAT0002816	UGAAACAUACACGGGAAACCUC
hsa-miR-495-3p	MIMAT0002817	AAACAAACAUGGUGCACUUCUU
hsa-miR-496	MIMAT0002818	UGAGUAUUACAUGGCCAAUCUC
hsa-miR-193b-3p	MIMAT0002819	AACUGGCCCUCAAAGUCCCGCU
hsa-miR-497-5p	MIMAT0002820	CAGCAGCACACUGUGGUUUGU
hsa-miR-181d-5p	MIMAT0002821	AACAUUCAUUGUUGUCGGUGGGU
hsa-miR-512-5p	MIMAT0002822	CACUCAGCCUUGAGGGCACUUUC
hsa-miR-512-3p	MIMAT0002823	AAGUGCUGUCAUAGCUGAGGUC
hsa-miR-498	MIMAT0002824	UUUCAAGCCAGGGGGCGUUUUUC
hsa-miR-520e	MIMAT0002825	AAAGUGCUUCCUUUUUGAGGG
hsa-miR-515-5p	MIMAT0002826	UUCUCCAAAAGAAAGCACUUUCUG
hsa-miR-515-3p	MIMAT0002827	GAGUGCCUUCUUUUGGAGCGUU
hsa-miR-519e-3p	MIMAT0002829	AAGUGCCUCCUUUUAGAGUGUU
hsa-miR-520f-3p	MIMAT0002830	AAGUGCUUCCUUUUAGAGGGUU
hsa-miR-519c-5p	MIMAT0002831	CUCUAGAGGGAAGCGCUUUCUG
hsa-miR-519c-3p	MIMAT0002832	AAAGUGCAUCUUUUUAGAGGAU
hsa-miR-520a-5p	MIMAT0002833	CUCCAGAGGGAAGUACUUUCU
hsa-miR-520a-3p	MIMAT0002834	AAAGUGCUUCCCUUUGGACUGU
hsa-miR-526b-5p	MIMAT0002835	CUCUUGAGGGAAGCACUUUCUGU
hsa-miR-519b-3p	MIMAT0002837	AAAGUGCAUCCUUUUAGAGGUU
hsa-miR-525-5p	MIMAT0002838	CUCCAGAGGGAUGCACUUUCU
hsa-miR-525-3p	MIMAT0002839	GAAGGCGCUUCCCUUUAGAGCG
hsa-miR-523-3p	MIMAT0002840	GAACGCGCUUCCCUAUAGAGGGU
hsa-miR-518f-3p	MIMAT0002842	GAAAGCGCUUCUCUUUAGAGG
hsa-miR-520b	MIMAT0002843	AAAGUGCUUCCUUUUAGAGGG
hsa-miR-518b	MIMAT0002844	CAAAGCGCUCCCCUUUAGAGGU
hsa-miR-526a	MIMAT0002845	CUCUAGAGGGAAGCACUUUCUG
hsa-miR-520c-3p	MIMAT0002846	AAAGUGCUUCCUUUUAGAGGGU
hsa-miR-518c-5p	MIMAT0002847	UCUCUGGAGGGAAGCACUUUCUG
hsa-miR-518c-3p	MIMAT0002848	CAAAGCGCUUCUCUUUAGAGUGU
hsa-miR-524-3p	MIMAT0002850	GAAGGCGCUUCCCUUUGGAGU
hsa-miR-517a-3p	MIMAT0002852	AUCGUGCAUCCCUUUAGAGUGU
hsa-miR-519d-3p	MIMAT0002853	CAAAGUGCCUCCCUUUAGAGUG
hsa-miR-521	MIMAT0002854	AACGCACUUCCCUUUAGAGUGU
hsa-miR-520d-5p	MIMAT0002855	CUACAAAGGGAAGCCCUUUC
hsa-miR-520d-3p	MIMAT0002856	AAAGUGCUUCUCUUUGGUGGGU

Official symbol	Accession number	Target Sequence
hsa-miR-517b-3p	MIMAT0002857	AUCGUGCAUCCCUUUAGAGUGU
hsa-miR-520g-3p	MIMAT0002858	ACAAAGUGCUUCCCUUUAGAGUGU
hsa-miR-516b-5p	MIMAT0002859	AUCUGGAGGUAAGAAGCACUUU
hsa-miR-516b-3p	MIMAT0002860	UGCUUCCUUUCAGAGGGU
hsa-miR-518e-3p	MIMAT0002861	AAAGCGCUUCCCUUCAGAGUG
hsa-miR-527	MIMAT0002862	CUGCAAAGGGAAGCCCUUUC
hsa-miR-518d-3p	MIMAT0002864	CAAAGCGCUUCCCUUUGGAGC
hsa-miR-517c-3p	MIMAT0002866	AUCGUGCAUCCUUUUAGAGUGU
hsa-miR-520h	MIMAT0002867	ACAAAGUGCUUCCCUUUAGAGU
hsa-miR-522-3p	MIMAT0002868	AAAAUGGUUCCCUUUAGAGUGU
hsa-miR-519a-3p	MIMAT0002869	AAAGUGCAUCCUUUUAGAGUGU
hsa-miR-499a-5p	MIMAT0002870	UUAAGACUUGCAGUGAUGUUU
hsa-miR-501-5p	MIMAT0002872	AAUCCUUUGUCCCUGGGUGAGA
hsa-miR-502-5p	MIMAT0002873	AUCCUUGCUAUCUGGGUGCUA
hsa-miR-503-5p	MIMAT0002874	UAGCAGCGGGAACAGUUCUGCAG
hsa-miR-504-5p	MIMAT0002875	AGACCCUGGUCUGCACUCUAUC
hsa-miR-505-3p	MIMAT0002876	CGUCAACACUUGCUGGUUUCCU
hsa-miR-513a-5p	MIMAT0002877	UUCACAGGGAGGUGUCAU
hsa-miR-506-3p	MIMAT0002878	UAAGGCACCCUUCUGAGUAGA
hsa-miR-507	MIMAT0002879	UUUUGCACCUUUUGGAGUGAA
hsa-miR-508-3p	MIMAT0002880	UGAUUGUAGCCUUUUGGAGUAGA
hsa-miR-509-3p	MIMAT0002881	UGAUUGGUACGUCUGUGGGUAG
hsa-miR-510-5p	MIMAT0002882	UACUCAGGAGAGUGGCAAUCAC
hsa-miR-514a-3p	MIMAT0002883	AUUGACACUUCUGUGAGUAGA
hsa-miR-532-5p	MIMAT0002888	CAUGCCUUGAGUGUAGGACCGU
hsa-miR-299-5p	MIMAT0002890	UGGUUUACCGUCCCACAUACAU
hsa-miR-455-5p	MIMAT0003150	UAUGUGCCUUUGGACUACAUCG
hsa-miR-493-3p	MIMAT0003161	UGAAGGUCUACUGUGUGCCAGG
hsa-miR-539-5p	MIMAT0003163	GGAGAAAUUAUCCUUGGUGUGU
hsa-miR-544a	MIMAT0003164	AUUCUGCAUUUUUAGCAAGUUC
hsa-miR-545-3p	MIMAT0003165	UCAGCAAACAUUUAUUGUGUGC
hsa-miR-487b-3p	MIMAT0003180	AAUCGUACAGGGUCAUCCACUU
hsa-miR-551a	MIMAT0003214	GCGACCCACUCUUGGUUUCCA
hsa-miR-552-3p	MIMAT0003215	AACAGGUGACUGGUUAGACAA
hsa-miR-553	MIMAT0003216	AAAACGGUGAGAUUUUGUUUU
hsa-miR-554	MIMAT0003217	GCUAGUCCUGACUCAGCCAGU
hsa-miR-92b-3p	MIMAT0003218	UAUUGCACUCGUCCCGGCCUCC

Official symbol	Accession number	Target Sequence
hsa-miR-555	MIMAT0003219	AGGGUAAGCUGAACCUCUGAU
hsa-miR-556-5p	MIMAT0003220	GAUGAGCUCAUUGUAAUAUGAG
hsa-miR-561-3p	MIMAT0003225	CAAAGUUUAAGAUCCUUGAAGU
hsa-miR-562	MIMAT0003226	AAAGUAGCUGUACCAUUUGC
hsa-miR-563	MIMAT0003227	AGGUUGACAUACGUUUCCC
hsa-miR-564	MIMAT0003228	AGGCACGGUGUCAGCAGGC
hsa-miR-566	MIMAT0003230	GGGCGCCUGUGAUCCCAAC
hsa-miR-567	MIMAT0003231	AGUAUGUUCUUCCAGGACAGAAC
hsa-miR-568	MIMAT0003232	AUGUAUAAAUGUAUACACAC
hsa-miR-551b-3p	MIMAT0003233	GCGACCCAUACUUGGUUUCAG
hsa-miR-570-3p	MIMAT0003235	CGAAAACAGCAAUUACCUUUGC
hsa-miR-571	MIMAT0003236	UGAGUUGGCCAUCUGAGUGAG
hsa-miR-572	MIMAT0003237	GUCCGCUCGGCGGUGGCCCA
hsa-miR-573	MIMAT0003238	CUGAAGUGAUGUGUAACUGAUCAG
hsa-miR-574-3p	MIMAT0003239	CACGCUCAUGCACACACCCACA
hsa-miR-575	MIMAT0003240	GAGCCAGUUGGACAGGAGC
hsa-miR-576-5p	MIMAT0003241	AUUCUAAUUUCUCCACGUCUUU
hsa-miR-577	MIMAT0003242	UAGAUAAAAUAUUGGUACCUG
hsa-miR-578	MIMAT0003243	CUUCUUGUGCUCUAGGAUUGU
hsa-miR-579-3p	MIMAT0003244	UUCAUUUGGUAUAAACCGCGAUU
hsa-miR-580-3p	MIMAT0003245	UUGAGAAUGAUGAAUCAUUAGG
hsa-miR-582-5p	MIMAT0003247	UUACAGUUGUUCAACCAGUUACU
hsa-miR-584-5p	MIMAT0003249	UUAUGGUUUGCCUGGGACUGAG
hsa-miR-585-3p	MIMAT0003250	UGGGCGUAUCUGUAUGCUA
hsa-miR-548a-3p	MIMAT0003251	CAAAACUGGCAAUUACUUUUGC
hsa-miR-587	MIMAT0003253	UUUCCAUAGGUGAUGAGUCAC
hsa-miR-548b-3p	MIMAT0003254	CAAGAACCUCAGUUGCUUUUGU
hsa-miR-590-5p	MIMAT0003258	GAGCUUAUUCAUAAAAGUGCAG
hsa-miR-591	MIMAT0003259	AGACCAUGGGUUCUCAUUGU
hsa-miR-592	MIMAT0003260	UUGUGUCAAUAUGCGAUGAUGU
hsa-miR-595	MIMAT0003263	GAAGUGUGCCGUGGUGUGUCU
hsa-miR-596	MIMAT0003264	AAGCCUGCCCGGCUCCUCGGG
hsa-miR-597-5p	MIMAT0003265	UGUGUCACUCGAUGACCACUGU
hsa-miR-598-3p	MIMAT0003266	UACGUCAUCGUUGUCAUCGUCA
hsa-miR-599	MIMAT0003267	GUUGUGUCAGUUUAUCAAAC
hsa-miR-600	MIMAT0003268	ACUUACAGACAAGAGCCUUGCUC
hsa-miR-601	MIMAT0003269	UGGUCUAGGAUUGUUGGAGGAG

Official symbol	Accession number	Target Sequence
hsa-miR-603	MIMAT0003271	CACACACUGCAAUUACUUUUGC
hsa-miR-604	MIMAT0003272	AGGCUGCGGAAUUCAGGAC
hsa-miR-605-5p	MIMAT0003273	UAAAUCCCAUGGUGCCUUCUCCU
hsa-miR-606	MIMAT0003274	AAACUACUGAAAAUCAAAGAU
hsa-miR-607	MIMAT0003275	GUUCAAAUCCAGAUCUAUAAC
hsa-miR-608	MIMAT0003276	AGGGGUGGUGUUGGGACAGCUCCGU
hsa-miR-610	MIMAT0003278	UGAGCUAAAUGUGUGCUGGGA
hsa-miR-612	MIMAT0003280	GCUGGGCAGGGCUUCUGAGCUCCUU
hsa-miR-613	MIMAT0003281	AGGAAUGUUCCUUCUUUGCC
hsa-miR-614	MIMAT0003282	GAACGCCUGUUCUUGCCAGGUGG
hsa-miR-615-3p	MIMAT0003283	UCCGAGCCUGGGUCUCCCUCUU
hsa-miR-617	MIMAT0003286	AGACUUCCCAUUUGAAGGUGGC
hsa-miR-619-3p	MIMAT0003288	GACCUGGACAUGUUUGUGCCCAGU
hsa-miR-620	MIMAT0003289	AUGGAGAUAGAUAUAGAAAU
hsa-miR-625-5p	MIMAT0003294	AGGGGGAAAGUUCUAUAGUCC
hsa-miR-626	MIMAT0003295	AGCUGUCUGAAAAUGUCUU
hsa-miR-627-5p	MIMAT0003296	GUGAGUCUCUAAGAAAAGAGGA
hsa-miR-628-3p	MIMAT0003297	UCUAGUAAGAGUGGCAGUCGA
hsa-miR-630	MIMAT0003299	AGUAUUCUGUACCAGGGAAGGU
hsa-miR-631	MIMAT0003300	AGACCUGGCCCAGACCUCAGC
hsa-miR-33b-5p	MIMAT0003301	GUGCAUUGCUGUUGCAUUGC
hsa-miR-637	MIMAT0003307	ACUGGGGGCUUUCGGGCUCUGCGU
hsa-miR-638	MIMAT0003308	AGGGAUCGCGGGCGGGUGGCGGCCU
hsa-miR-639	MIMAT0003309	AUCGCUGCGGUUGCGAGCGCUGU
hsa-miR-640	MIMAT0003310	AUGAUCCAGGAACCUGCCUCU
hsa-miR-641	MIMAT0003311	AAAGACAUAGGAUAGAGUCACCUC
hsa-miR-642a-5p	MIMAT0003312	GUCCCUCUCCAAAUGUGUCUUG
hsa-miR-643	MIMAT0003313	ACUUGUAUGCUAGCUCAGGUAG
hsa-miR-644a	MIMAT0003314	AGUGUGGCUUUCUUAGAGC
hsa-miR-648	MIMAT0003318	AAGUGUGCAGGGCACUGGU
hsa-miR-649	MIMAT0003319	AAACCUGUGUUGUUCAAGAGUC
hsa-miR-650	MIMAT0003320	AGGAGGCAGCGCUCUCAGGAC
hsa-miR-651-5p	MIMAT0003321	UUUAGGAUAAGCUUGACUUUUG
hsa-miR-652-3p	MIMAT0003322	AAUGGCGCCACUAGGGUUGUG
hsa-miR-548d-3p	MIMAT0003323	CAAAAACCACAGUUUCUUUUGC
hsa-miR-661	MIMAT0003324	UGCCUGGGUCUCUGGCCUGCGCGU
hsa-miR-663a	MIMAT0003326	AGGCGGGGCGCCGCGGGACCGC

Official symbol	Accession number	Target Sequence
hsa-miR-449b-5p	MIMAT0003327	AGGCAGUGUAUUGUUAGCUGGC
hsa-miR-411-5p	MIMAT0003329	UAGUAGACCGUAUAGCGUACG
hsa-miR-654-5p	MIMAT0003330	UGGUGGGCCGCAGAACAUGUGC
hsa-miR-655-3p	MIMAT0003331	AUAAUACAUGGUUAACCUCUUU
hsa-miR-656-3p	MIMAT0003332	AAUAUUAUACAGUCAACCUCU
hsa-miR-549a	MIMAT0003333	UGACAACUAUGGAUGAGCUCU
hsa-miR-660-5p	MIMAT0003338	UACCCAUUGCAUAUCGGAGUUG
hsa-miR-421	MIMAT0003339	AUCAACAGACAUUAAUUGGGCGC
hsa-miR-542-5p	MIMAT0003340	UCGGGGAUCAUCAUGUCACGAGA
hsa-miR-363-5p	MIMAT0003385	CGGGUGGAUCACGAUGCAAUUU
hsa-miR-542-3p	MIMAT0003389	UGUGACAGAUUGAUAACUGAAA
hsa-miR-425-5p	MIMAT0003393	AAUGACACGAUCACUCCCGUUGA
hsa-miR-758-3p	MIMAT0003879	UUUGUGACCUGGUCCACUAACC
hsa-miR-671-5p	MIMAT0003880	AGGAAGCCCUGGAGGGGCUGGAG
hsa-miR-767-5p	MIMAT0003882	UGCACCAUGGUUGUCUGAGCAUG
hsa-miR-767-3p	MIMAT0003883	UCUGCUCAUACCCCAUGGUUUCU
hsa-miR-454-3p	MIMAT0003885	UAGUGCAAUAUUGCUUAUAGGGU
hsa-miR-769-5p	MIMAT0003886	UGAGACCUCUGGGUUCUGAGCU
hsa-miR-769-3p	MIMAT0003887	CUGGGAUCUCCGGGGUCUUGGUU
hsa-miR-766-3p	MIMAT0003888	ACUCCAGCCCCACAGCCUCAGC
hsa-miR-765	MIMAT0003945	UGGAGGAGAAGGAAGGUGAUG
hsa-miR-770-5p	MIMAT0003948	UCCAGUACCACGUGUCAGGGCCA
hsa-miR-802	MIMAT0004185	CAGUAACAAAGAUUCAUCCUUGU
hsa-miR-675-5p	MIMAT0004284	UGGUGCGGAGAGGGCCCACAGUG
hsa-miR-297	MIMAT0004450	AUGUAUGUGUGCAUGUGCAUG
hsa-miR-25-5p	MIMAT0004498	AGGCGGAGACUUGGGCAAUUG
hsa-miR-28-3p	MIMAT0004502	CACUAGAUUGUGAGCUCCUGGA
hsa-miR-92a-1-5p	MIMAT0004507	AGGUUGGGAUCGGUUGCAAUGCU
hsa-miR-139-3p	MIMAT0004552	UGGAGACGCGGCCCUGUUGGAGU
hsa-miR-181a-2-3p	MIMAT0004558	ACCACUGACCGUUGACUGUACC
hsa-miR-196a-3p	MIMAT0004562	CGGCAACAAGAAACUGCCUGAG
hsa-miR-199b-3p	MIMAT0004563	ACAGUAGUCUGCACAUUGGUUA
hsa-miR-219a-1-3p	MIMAT0004567	AGAGUUGAGUCUGGACGUCCCG
hsa-miR-221-5p	MIMAT0004568	ACCUGGCAUACAAUGUAGAUUU
hsa-miR-140-3p	MIMAT0004597	UACCACAGGGUAGAACCACGG

Official symbol	Accession number	Target Sequence
hsa-miR-125a-3p	MIMAT0004602	ACAGGUGAGGUUCUUGGGAGCC
hsa-miR-127-5p	MIMAT0004604	CUGAAGCUCAGAGGGCUCUGAU
hsa-miR-129-2-3p	MIMAT0004605	AAGCCCUUACCCCAAAAAGCAU
hsa-miR-188-3p	MIMAT0004613	CUCCCACAUGCAGGGUUUGCA
hsa-miR-193a-5p	MIMAT0004614	UGGGUCUUUGCGGGCGAGAUGA
hsa-miR-30c-1-3p	MIMAT0004674	CUGGGAGAGGGUUGUUUACUCC
hsa-miR-219a-2-3p	MIMAT0004675	AGAAUUGUGGCUGGACAUCUGU
hsa-miR-34b-3p	MIMAT0004676	CAAUCACUAACUCCACUGCCAU
hsa-miR-34c-3p	MIMAT0004677	AAUCACUAACCACACGGCCAGG
hsa-miR-296-3p	MIMAT0004679	GAGGGUUGGGUGGAGGCUCUCC
hsa-miR-361-3p	MIMAT0004682	UCCCCCAGGUGUGAUUCUGAUUU
hsa-miR-362-3p	MIMAT0004683	AACACACCUAUUCAAGGAUUCA
hsa-miR-371a-5p	MIMAT0004687	ACUCAAACUGUGGGGGCACU
hsa-miR-374a-3p	MIMAT0004688	CUUAUCAGAUUGUAUUGUAAUU
hsa-miR-340-5p	MIMAT0004692	UUAUAAAGCAAUGAGACUGAUU
hsa-miR-330-5p	MIMAT0004693	UCUCUGGGCCUGUGUCUUAGGC
hsa-miR-342-5p	MIMAT0004694	AGGGGUGCUAUCUGUGAUUGA
hsa-miR-337-5p	MIMAT0004695	GAACGGCUUCAUACAGGAGUU
hsa-miR-323a-5p	MIMAT0004696	AGGUGGUCCGUGGCGCGUUCGC
hsa-miR-151a-5p	MIMAT0004697	UCGAGGAGCUCACAGUCUAGU
hsa-miR-331-5p	MIMAT0004700	CUAGGUAUGGUCCCAGGGAUCC
hsa-miR-338-5p	MIMAT0004701	AACAAUAUCCUGGUGCUGAGUG
hsa-miR-339-3p	MIMAT0004702	UGAGCGCCUCGACGACAGAGCCG
hsa-miR-423-5p	MIMAT0004748	UGAGGGGCAGAGAGCGAGACUUU
hsa-miR-483-5p	MIMAT0004761	AAGACGGGAGGAAAGAAGGGAG
hsa-miR-486-3p	MIMAT0004762	CGGGGCAGCUCAGUACAGGAU
hsa-miR-488-3p	MIMAT0004763	UUGAAAGGCUAUUUCUUGGUC
hsa-miR-490-5p	MIMAT0004764	CCAUGGAUCUCCAGGUGGGU
hsa-miR-491-3p	MIMAT0004765	CUUAUGCAAGAUUCCCUUCUAC
hsa-miR-146b-3p	MIMAT0004766	UGCCCUGUGGACUCAGUUCUGG
hsa-miR-193b-5p	MIMAT0004767	CGGGGUUUUGAGGGCGAGAUGA
hsa-miR-516a-5p	MIMAT0004770	UUCUCGAGGAAAGAAGCACUUUC
hsa-miR-499a-3p	MIMAT0004772	AACAUCACAGCAAGUCUGUGCU
hsa-miR-500a-5p	MIMAT0004773	UAAUCCUUGCUACCUGGGUGAGA
hsa-miR-501-3p	MIMAT0004774	AAUGCACCCGGGCAAGGAUUCU
hsa-miR-502-3p	MIMAT0004775	AAUGCACCUGGGCAAGGAUUCA

Official symbol	Accession number	Target Sequence
hsa-miR-513a-3p	MIMAT0004777	UAAAUUUCACCUUUCUGAGAAGG
hsa-miR-508-5p	MIMAT0004778	UACUCCAGAGGGCGUCACUCAUG
hsa-miR-509-5p	MIMAT0004779	UACUGCAGACAGUGGCAAUCA
hsa-miR-532-3p	MIMAT0004780	CCUCCCACACCCAAGGCUUGCA
hsa-miR-455-3p	MIMAT0004784	GCAGUCCAUGGGCAUAUACAC
hsa-miR-556-3p	MIMAT0004793	AUAUUACCAUUAGCUCAUCUUU
hsa-miR-574-5p	MIMAT0004795	UGAGUGUGUGUGUGUGAGUGUGU
hsa-miR-576-3p	MIMAT0004796	AAGAUGUGGAAAAAUUGGAAUC
hsa-miR-582-3p	MIMAT0004797	UAACUGGUUGAACAACUGAACC
hsa-miR-589-5p	MIMAT0004799	UGAGAACCACGUCUGCUCUGAG
hsa-miR-550a-5p	MIMAT0004800	AGUGCCUGAGGGAGUAAGAGCCC
hsa-miR-590-3p	MIMAT0004801	UAAUUUUAUGUAUAAGCUAGU
hsa-miR-593-3p	MIMAT0004802	UGUCUCUGCUGGGGUUUCU
hsa-miR-548a-5p	MIMAT0004803	AAAAGUAAUUGCGAGUUUUACC
hsa-miR-615-5p	MIMAT0004804	GGGGGUCCCCGGUGCUCGGAUC
hsa-miR-616-3p	MIMAT0004805	AGUCAUUGGAGGGUUUGAGCAG
hsa-miR-548c-5p	MIMAT0004806	AAAAGUAAUUGCGGUUUUUGCC
hsa-miR-624-3p	MIMAT0004807	CACAAGGUAUUGGUAUUACCU
hsa-miR-628-5p	MIMAT0004809	AUGCUGACAUAUUUACUAGAGG
hsa-miR-629-5p	MIMAT0004810	UGGGUUUACGUUGGGAGAACU
hsa-miR-548d-5p	MIMAT0004812	AAAAGUAAUUGUGGUUUUUGCC
hsa-miR-411-3p	MIMAT0004813	UAUGUAACACGGUCCACUAACC
hsa-miR-654-3p	MIMAT0004814	UAUGUCUGCUGACCAUCACCUU
hsa-miR-671-3p	MIMAT0004819	UCCGGUUCUCAGGGCUCCACC
hsa-miR-298	MIMAT0004901	AGCAGAAGCAGGGAGGUUCUCCCA
hsa-miR-891a-5p	MIMAT0004902	UGCAACGAACCUGAGCCACUGA
hsa-miR-300	MIMAT0004903	UAUACAAGGGCAGACUCUCUCU
hsa-miR-892a	MIMAT0004907	CACUGUGUCCUUUCUGCGUAG
hsa-miR-450b-5p	MIMAT0004909	UUUUGCAAUAUGUUCCUGAAUA
hsa-miR-450b-3p	MIMAT0004910	UUGGGAUCAUUUUGCAUCCAUA
hsa-miR-874-3p	MIMAT0004911	CUGCCCUGGCCCGAGGGACCGA
hsa-miR-890	MIMAT0004912	UACUUGGAAAGGCAUCAGUUG
hsa-miR-891b	MIMAT0004913	UGCAACUUACCUGAGUCAUUGA
hsa-miR-888-5p	MIMAT0004916	UACUCAAAAAGCUGUCAGUCA
hsa-miR-892b	MIMAT0004918	CACUGGCUCCUUUCUGGGUAGA
hsa-miR-541-3p	MIMAT0004920	UGGUGGGCACAGAAUCUGGACU
hsa-miR-889-3p	MIMAT0004921	UUAAUAUCGGACAACCAUUGU

Official symbol	Accession number	Target Sequence
hsa-miR-875-3p	MIMAT0004923	CCUGGAAACACUGAGGUUGUG
hsa-miR-876-5p	MIMAT0004924	UGGAUUUCUUUGUGAAUCACCA
hsa-miR-876-3p	MIMAT0004925	UGGUGGUUUACAAAGUAAUUCA
hsa-miR-708-5p	MIMAT0004926	AAGGAGCUUACAAUCUAGCUGGG
hsa-miR-147b	MIMAT0004928	GUGUGCGGAAAUGCUUCUGCUA
hsa-miR-190b	MIMAT0004929	UGAUAUGUUUGAUAUUGGGUU
hsa-miR-744-5p	MIMAT0004945	UGCGGGGCUAGGGCUAACAGCA
hsa-miR-885-5p	MIMAT0004947	UCCAUUACACUACCCUGCCUCU
hsa-miR-885-3p	MIMAT0004948	AGGCAGCGGGGUGUAGUGGAUA
hsa-miR-877-5p	MIMAT0004949	GUAGAGGAGAUGGCGCAGGG
hsa-miR-887-3p	MIMAT0004951	GUGAACGGGCGCCAUCCCGAGG
hsa-miR-665	MIMAT0004952	ACCAGGAGGCUGAGGCCCCU
hsa-miR-873-5p	MIMAT0004953	GCAGGAACUUGUGAGUCUCCU
hsa-miR-543	MIMAT0004954	AAACAUUCGCGGUGCACUUCUU
hsa-miR-374b-5p	MIMAT0004955	AUAUAAUACAACCUGCUAAGUG
hsa-miR-760	MIMAT0004957	CGGCUCUGGGUCUGUGGGGA
hsa-miR-301b-3p	MIMAT0004958	CAGUGCAAUGAUAUUGUCAAAGC
hsa-miR-216b-5p	MIMAT0004959	AAAUCUCUGCAGGCAAAUGUGA
hsa-miR-208b-3p	MIMAT0004960	AUAAGACGAACAAAAGGUUUGU
hsa-miR-922	MIMAT0004972	GCAGCAGAGAAUAGGACUACGUC
hsa-miR-924	MIMAT0004974	AGAGUCUUGUGAUGUCUUGC
hsa-miR-509-3-5p	MIMAT0004975	UACUGCAGACGUGGCAAUCAUG
hsa-miR-933	MIMAT0004976	UGUGCGCAGGGAGACCUCUCCC
hsa-miR-934	MIMAT0004977	UGUCUACUACUGGAGACACUGG
hsa-miR-935	MIMAT0004978	CCAGUUACCGCUUCCGCUACCGC
hsa-miR-936	MIMAT0004979	ACAGUAGAGGGAGGAAUCGCAG
hsa-miR-937-3p	MIMAT0004980	AUCCGCGCUCUGACUCUCUGCC
hsa-miR-939-5p	MIMAT0004982	UGGGGAGCUGAGGCUCUGGGGGUG
hsa-miR-940	MIMAT0004983	AAGGCAGGGCCCCCGCUCCCC
hsa-miR-941	MIMAT0004984	CACCCGGCUGUGUGCACAUGUGC
hsa-miR-942-5p	MIMAT0004985	UCUUCUCUGUUUUGGCCAUGUG
hsa-miR-944	MIMAT0004987	AAAUUAUUGUACAUCGGAUGAG
hsa-miR-523-5p	MIMAT0005449	CUCUAGAGGGAAGCGCUUUCUG
hsa-miR-518e-5p	MIMAT0005450	CUCUAGAGGGAAGCGCUUUCUG
hsa-miR-522-5p	MIMAT0005451	CUCUAGAGGGAAGCGCUUUCUG
hsa-miR-519a-5p	MIMAT0005452	CUCUAGAGGGAAGCGCUUUCUG
hsa-miR-519b-5p	MIMAT0005454	CUCUAGAGGGAAGCGCUUUCUG

Official symbol	Accession number	Target Sequence
hsa-miR-520c-5p	MIMAT0005455	CUCUAGAGGGAAGCACUUUCUG
hsa-miR-518d-5p	MIMAT0005456	CUCUAGAGGGAAGCACUUUCUG
hsa-miR-518a-5p	MIMAT0005457	CUGCAAAGGGAAGCCCUUUC
hsa-miR-1224-5p	MIMAT0005458	GUGAGGACUCGGGAGGUGG
hsa-miR-1224-3p	MIMAT0005459	CCCCACCUCCUCUCUCCUCAG
hsa-miR-1226-3p	MIMAT0005577	UCACCAGCCCUGUGUUCCCUAG
hsa-miR-1228-3p	MIMAT0005583	UCACACCUGCCUCGCCCCCC
hsa-miR-1233-3p	MIMAT0005588	UGAGCCCUGUCCUCCCGCAG
hsa-miR-1234-3p	MIMAT0005589	UCGGCCUGACCACCCACCCCAC
hsa-miR-1236-3p	MIMAT0005591	CCUCUUCCCCUUGUCUCUCCAG
hsa-miR-513b-5p	MIMAT0005788	UUCACAAGGAGGUGUCAUUUAU
hsa-miR-513c-5p	MIMAT0005789	UUCUCAAGGAGGUGUCGUUUAU
hsa-miR-1264	MIMAT0005791	CAAGUCUUAUUUGAGCACCUGUU
hsa-miR-320b	MIMAT0005792	AAAAGCUGGGUUGAGAGGGCAA
hsa-miR-320c	MIMAT0005793	AAAAGCUGGGUUGAGAGGGU
hsa-miR-1296-5p	MIMAT0005794	UUAGGGCCCUGGCUCCAUCUCC
hsa-miR-1323	MIMAT0005795	UCAAAACUGAGGGGCAUUUUCU
hsa-miR-1271-5p	MIMAT0005796	CUUGGCACCUAGCAAGCACUCA
hsa-miR-1301-3p	MIMAT0005797	UUGCAGCUGCCUGGGAGUGACUUC
hsa-miR-1185-5p	MIMAT0005798	AGAGGAUACCCUUUGUAUGUU
hsa-miR-1283	MIMAT0005799	UCUACAAAGGAAAGCGCUUUCU
hsa-miR-1298-5p	MIMAT0005800	UUCAUUCGGCUGUCCAGAUGUA
hsa-miR-1178-3p	MIMAT0005823	UUGCUCACUGUUCUUCCCUAG
hsa-miR-1180-3p	MIMAT0005825	UUUCCGGCUCGCGUGGGUGUGU
hsa-miR-1183	MIMAT0005828	CACUGUAGGUGAUGGUGAGAGUGGGCA
hsa-miR-1200	MIMAT0005863	CUCCUGAGCCAUUCUGAGCCUC
hsa-miR-1202	MIMAT0005865	GUGCCAGCUGCAGUGGGGGAG
hsa-miR-1203	MIMAT0005866	CCCGGAGCCAGGAUGCAGCUC
hsa-miR-1204	MIMAT0005868	UCGUGGCCUGGUCUCCAUUAU
hsa-miR-1205	MIMAT0005869	UCUGCAGGGUUUGCUUUGAG
hsa-miR-1206	MIMAT0005870	UGUUCAUGUAGAUGUUUAAGC
hsa-miR-548e-3p	MIMAT0005874	AAAAACUGAGACUACUUUUGCA
hsa-miR-548j-5p	MIMAT0005875	AAAAGUAAUUGCGGUCUUUGGU
hsa-miR-1285-3p	MIMAT0005876	UCUGGGCAACAAAGUGAGACCU
hsa-miR-1286	MIMAT0005877	UGCAGGACCAAGAUGAGCCCU
hsa-miR-1287-5p	MIMAT0005878	UGCUGGAUCAGUGGUUCGAGUC
hsa-miR-1289	MIMAT0005879	UGGAGUCCAGGAAUCUGCAUUUU

Official symbol	Accession number	Target Sequence
hsa-miR-1290	MIMAT0005880	UGGAUUUUUGGAUCAGGGA
hsa-miR-1291	MIMAT0005881	UGGCCCUGACUGAAGACCAGCAGU
hsa-miR-548k	MIMAT0005882	AAAAGUACUUGCGGAUUUUGCU
hsa-miR-1293	MIMAT0005883	UGGGUGGUCUGGAGAUUUGUGC
hsa-miR-1295a	MIMAT0005885	UUAGGCCGCAGAUCUGGGUGA
hsa-miR-1297	MIMAT0005886	UUCAAGUAAUUCAGGUG
hsa-miR-1299	MIMAT0005887	UUCUGGAAUUCUGUGUGAGGGA
hsa-miR-548l	MIMAT0005889	AAAAGUAUUUGCGGGUUUUGUC
hsa-miR-1302	MIMAT0005890	UUGGGACAUACUUAUGCUAAA
hsa-miR-1303	MIMAT0005891	UUUAGAGACGGGGUCUUGCUCU
hsa-miR-1304-5p	MIMAT0005892	UUUGAGGCUACAGUGAGAUGUG
hsa-miR-1305	MIMAT0005893	UUUUCAACUCUAAUGGGAGAGA
hsa-miR-1244	MIMAT0005896	AAGUAGUUGGUUUGUAUGAGAUGGUU
hsa-miR-1245a	MIMAT0005897	AAGUGAUCUAAAGGCCUACAU
hsa-miR-1246	MIMAT0005898	AAUGGAUUUUUGGAGCAGG
hsa-miR-1247-5p	MIMAT0005899	ACCCGUCCCGUUCGUCCCCGGA
hsa-miR-1248	MIMAT0005900	ACCUUCUUGUAUAAGCACUGUGCUAAA
hsa-miR-1249-3p	MIMAT0005901	ACGCCCUUCCCCCCCUUCUUCA
hsa-miR-1250-5p	MIMAT0005902	ACGGUGCUGGAUGUGGCCUUU
hsa-miR-1253	MIMAT0005904	AGAGAAGAAGAUCAGCCUGCA
hsa-miR-1254	MIMAT0005905	AGCCUGGAAGCUGGAGCCUGCAGU
hsa-miR-1255a	MIMAT0005906	AGGAUGAGCAAAGAAAGUAGAUU
hsa-miR-1257	MIMAT0005908	AGUGAAUGAUGGGUUCUGACC
hsa-miR-1258	MIMAT0005909	AGUUAGGAUUAGGUCGUGGAA
hsa-miR-1260a	MIMAT0005911	AUCCCACCUCUGCCACCA
hsa-miR-548g-3p	MIMAT0005912	AAAACUGUAAUUACUUUUGUAC
hsa-miR-1261	MIMAT0005913	AUGGAUAAGGCUUUGGCUU
hsa-miR-1262	MIMAT0005914	AUGGGUGAAUUUGUAGAAGGAU
hsa-miR-548n	MIMAT0005916	CAAAAGUAAUUGUGGAUUUUGU
hsa-miR-548m	MIMAT0005917	CAAAGGUAUUUGUGGUUUUUG
hsa-miR-548o-3p	MIMAT0005919	CCAAAACUGCAGUUACUUUUGC
hsa-miR-1266-5p	MIMAT0005920	CCUCAGGGCUGUAGAACAGGGCU
hsa-miR-1268a	MIMAT0005922	CGGGCGUGGUGGUGGGGG
hsa-miR-1269a	MIMAT0005923	CUGGACUGAGCCGUGCUACUGG
hsa-miR-1270	MIMAT0005924	CUGGAGAUAUGGAAGAGCUGUGU
hsa-miR-1272	MIMAT0005925	GAUGAUGAUGGCAGCAAAUUCUGAAA
hsa-miR-548h-5p	MIMAT0005928	AAAAGUAAUCGCGGUUUUUGUC

Official symbol	Accession number	Target Sequence
hsa-miR-1275	MIMAT0005929	GUGGGGGAGAGGCUGUC
hsa-miR-1276	MIMAT0005930	UAAAGAGCCCUGUGGAGACA
hsa-miR-302e	MIMAT0005931	UAAGUGCUUCCAUGCUU
hsa-miR-302f	MIMAT0005932	UAAUUGCUUCCAUGUUU
hsa-miR-1277-3p	MIMAT0005933	UACGUAGAUAUAUAUGUAUUUU
hsa-miR-548i	MIMAT0005935	AAAAGUAAUUGCGGAUUUUGCC
hsa-miR-1278	MIMAT0005936	UAGUACUGUGCAUAUCAUCUAU
hsa-miR-1279	MIMAT0005937	UCAUAUUGCUUCUUUCU
hsa-miR-1281	MIMAT0005939	UCGCCUCCUCCUCUCCC
hsa-miR-1288-3p	MIMAT0005942	UGGACUGCCCUGAUCUGGAGA
hsa-miR-1252-5p	MIMAT0005944	AGAAGGAAAUUGAAUUCAUUUA
hsa-miR-1255b-5p	MIMAT0005945	CGGAUGAGCAAAGAAAGUGGUU
hsa-miR-664a-3p	MIMAT0005949	UAUUCAUUUAUCCCCAGCCUACA
hsa-miR-1306-3p	MIMAT0005950	ACGUUGGCUCUGGUGGUG
hsa-miR-1307-3p	MIMAT0005951	ACUCGGCGUGGCGUCGGUCGUG
hsa-miR-1322	MIMAT0005953	GAUGAUGCUGCUGAUGCUG
hsa-miR-1197	MIMAT0005955	UAGGACACAUGGUCUACUUCU
hsa-miR-320d	MIMAT0006764	AAAAGCUGGGUUGAGAGGA
hsa-miR-1827	MIMAT0006767	UGAGGCAGUAGAUUGAAU
hsa-miR-516a-3p	MIMAT0006778	UGCUUCCUUUCAGAGGGU
hsa-miR-1469	MIMAT0007347	CUCGGCGCGGGGCGCGGGCUCC
hsa-miR-1537-3p	MIMAT0007399	AAAACCGUCUAGUUACAGUUGU
hsa-miR-1908-5p	MIMAT0007881	CGGCGGGGACGGCGAUUGGUC
hsa-miR-1909-3p	MIMAT0007883	CGCAGGGGCCGGGUGCUCACCG
hsa-miR-1910-5p	MIMAT0007884	CCAGUCCUGUGCCUGCCGCCU
hsa-miR-1915-3p	MIMAT0007892	CCCCAGGGCGACGCGGCGGG
hsa-miR-2113	MIMAT0009206	AUUUGUGCUUGGCUCUGUCAC
hsa-miR-1972	MIMAT0009447	UCAGGCCAGGCACAGUGGCUCA
hsa-miR-1973	MIMAT0009448	ACCGUGCAAAGGUAGCAUA
hsa-miR-1976	MIMAT0009451	CCUCCUGCCCUCCUUGCUGU
hsa-miR-2053	MIMAT0009978	GUGUUAAUUAAACCUCUAUUUAC
hsa-miR-2110	MIMAT0010133	UUGGGGAAACGGCCGCUGAGUG
hsa-miR-151b	MIMAT0010214	UCGAGGAGCUCACAGUCU
hsa-miR-449c-5p	MIMAT0010251	UAGGCAGUGUAUUGCUAGCGGCUGU
hsa-miR-761	MIMAT0010364	GCAGCAGGGUGAAACUGACACA
hsa-miR-764	MIMAT0010367	GCAGGUGCUCACUUGUCCUCCU

Official symbol	Accession number	Target Sequence
hsa-miR-2116-5p	MIMAT0011160	GGUUCUUAGCAUAGGAGGUCU
hsa-miR-2117	MIMAT0011162	UGUUCUCUUUGCCAAGGACAG
hsa-miR-548q	MIMAT0011163	GCUGGUGCAAAAGUAAUGGCGG
hsa-miR-2278	MIMAT0011778	GAGAGCAGUGUGUGUUGCCUGG
hsa-miR-2682-5p	MIMAT0013517	CAGGCAGUGACUGUUCAGACGUC
hsa-miR-3127-5p	MIMAT0014990	AUCAGGGCUUGUGGAAUGGGAAG
hsa-miR-3130-3p	MIMAT0014994	GCUGCACCGGAGACUGGGUAA
hsa-miR-3131	MIMAT0014996	UCGAGGACUGGUGGAAGGGCCUU
hsa-miR-378b	MIMAT0014999	ACUGGACUUGGAGGCAGAA
hsa-miR-3136-5p	MIMAT0015003	CUGACUGAAUAGGUAGGGUCAUU
hsa-miR-3140-3p	MIMAT0015008	AGCUUUUGGGAAUUCAGGUAGU
hsa-miR-3144-5p	MIMAT0015014	AGGGGACCAAAGAGAUAUAUAG
hsa-miR-3144-3p	MIMAT0015015	AUAUACCUGUUCGGUCUCUUUA
hsa-miR-1273c	MIMAT0015017	GGCGACAAAACGAGACCCUGUC
hsa-miR-3147	MIMAT0015019	GGUUGGGCAGUGAGGAGGGUGUGA
hsa-miR-548v	MIMAT0015020	AGCUACAGUUACUUUUGCACCA
hsa-miR-3151-5p	MIMAT0015024	GGUGGGGCAAUGGGAUCAGGU
hsa-miR-3074-3p	MIMAT0015027	GAUAUCAGCUCAGUAGGCACCG
hsa-miR-3158-3p	MIMAT0015032	AAGGGCUUCCUCUCUGCAGGAC
hsa-miR-3161	MIMAT0015035	CUGAUAAGAACAGAGGCCCAGAU
hsa-miR-3164	MIMAT0015038	UGUGACUUUAAGGGAAAUGGCG
hsa-miR-1260b	MIMAT0015041	AUCCCACCACUGCCACCAU
hsa-miR-3168	MIMAT0015043	GAGUUCUACAGUCAGAC
hsa-miR-1193	MIMAT0015049	GGGAUGGUAGACCGGUGACGUGC
hsa-miR-323b-3p	MIMAT0015050	CCCAAUACACGGUCGACCUCUU
hsa-miR-3179	MIMAT0015056	AGAAGGGGUGAAAUUUAAACGU
hsa-miR-3180-5p	MIMAT0015057	CUUCCAGACGCUCCGCCCCACGUCG
hsa-miR-3180-3p	MIMAT0015058	UGGGGCGGAGCUUCCGGAGGCC
hsa-miR-3182	MIMAT0015062	GCUUCUGUAGUGUAGUC
hsa-miR-3185	MIMAT0015065	AGAAGAAGGCGGUCGGUCUGCGG
hsa-miR-3065-5p	MIMAT0015066	UCAACAAAAUCACUGAUGCUGGA
hsa-miR-320e	MIMAT0015072	AAAGCUGGGUUGAGAAGG
hsa-miR-3192-5p	MIMAT0015076	UCUGGGAGGUUGUAGCAGUGGAA
hsa-miR-3195	MIMAT0015079	CGCGCCGGGCCCGGGUU
hsa-miR-3196	MIMAT0015080	CGGGGCGGCAGGGGCCUC
hsa-miR-514b-5p	MIMAT0015087	UUCUCAAGAGGGAGGCAAUCAU
hsa-miR-514b-3p	MIMAT0015088	AUUGACACCUCUGUGAGUGGA

Official symbol	Accession number	Target Sequence
hsa-miR-3202	MIMAT0015089	UGGAAGGGAGAAGAGCUUUAAU
hsa-miR-3065-3p	MIMAT0015378	UCAGCACCAGGAUAUUGUUGGAG
hsa-miR-378c	MIMAT0016847	ACUGGACUUGGAGUCAGAAGAGUGG
hsa-miR-4284	MIMAT0016915	GGGCUCACAUCACCCCAU
hsa-miR-4286	MIMAT0016916	ACCCCACUCCUGGUACC
hsa-miR-3605-5p	MIMAT0017981	UGAGGAUGGAUAGCAAGGAAGCC
hsa-miR-3605-3p	MIMAT0017982	CCUCCGUGUUACCUGUCCUCUAG
hsa-miR-3613-5p	MIMAT0017990	UGUUGUACUUUUUUUUUUGUUC
hsa-miR-3613-3p	MIMAT0017991	ACAAAAAAAAAAGCCCAACCCUUC
hsa-miR-3614-5p	MIMAT0017992	CCACUUGGAUCUGAAGGCUGCCC
hsa-miR-3614-3p	MIMAT0017993	UAGCCUUCAGAUCUUGGUGUUUU
hsa-miR-3615	MIMAT0017994	UCUCUCGGCUCCUCGCGGCUC
hsa-miR-23c	MIMAT0018000	AUCACAUUGCCAGUGAUUACCC
hsa-miR-3690	MIMAT0018119	ACCUGGACCCAGCGUAGACAAAG
hsa-miR-3180	MIMAT0018178	UGGGGCGGAGCUUCCGGAG
hsa-miR-3916	MIMAT0018190	AAGAGGAAGAAAUGGCUGGUUCUCAG
hsa-miR-3918	MIMAT0018192	ACAGGGCCGCAGAUGGAGACU
hsa-miR-3150b-3p	MIMAT0018194	UGAGGAGAUCGUCGAGGUUGG
hsa-miR-3928-3p	MIMAT0018205	GGAGGAACCUUGGAGCUUCGGC
hsa-miR-3934-5p	MIMAT0018349	UCAGGUGUGGAAACUGAGGCAG
hsa-miR-548y	MIMAT0018354	AAAAGUAAUCACUGUUUUUGCC
hsa-miR-374c-5p	MIMAT0018443	AUAAUACAACCUGCUAAGUGCU
hsa-miR-548z	MIMAT0018446	CAAAAACCGCAAUUACUUUUGCA
hsa-miR-548aa	MIMAT0018447	AAAAACCACAAUUACUUUUGCACCA
hsa-miR-1268b	MIMAT0018925	CGGGCGUGGUGGUGGGGGUG
hsa-miR-378d	MIMAT0018926	ACUGGACUUGGAGUCAGAAA
hsa-miR-378e	MIMAT0018927	ACUGGACUUGGAGUCAGGA
hsa-miR-378f	MIMAT0018932	ACUGGACUUGGAGCCAGAAG
hsa-miR-4421	MIMAT0018934	ACCUGUCUGUGGAAAGGAGCUA
hsa-miR-378g	MIMAT0018937	ACUGGGCUUGGAGUCAGAAG
hsa-miR-4425	MIMAT0018940	UGUUGGGAUUCAGCAGGACCAU
hsa-miR-548ad-3p	MIMAT0018946	GAAAACGACAAUGACUUUUGCA
hsa-miR-4431	MIMAT0018947	GCGACUCUGAAAACUAGAAGGU
hsa-miR-4435	MIMAT0018951	AUGGCCAGAGCUCACACAGAGG
hsa-miR-4443	MIMAT0018961	UUGGAGGCGUGGGUUUU
hsa-miR-4448	MIMAT0018967	GGCUCCUUGGUCUAGGGGUA

Official symbol	Accession number	Target Sequence
hsa-miR-548ah-5p	MIMAT0018972	AAAAGUGAUUGCAGUGUUUG
hsa-miR-4451	MIMAT0018973	UGGUAGAGCUGAGGACA
hsa-miR-4454	MIMAT0018976	GGAUCCGAGUCACGGCACCA
hsa-miR-4455	MIMAT0018977	AGGGUGUGUGUGUUUUU
hsa-miR-4458	MIMAT0018980	AGAGGUAGGUGUGGAAGAA
hsa-miR-4461	MIMAT0018983	GAUUGAGACUAGUAGGGCUAGGC
hsa-miR-378h	MIMAT0018984	ACUGGACUUGGUGUCAGAUGG
hsa-miR-548ai	MIMAT0018989	AAAGGUAAUUGCAGUUUUUCCC
hsa-miR-548ak	MIMAT0019013	AAAAGUAACUGCGGUUUUUGA
hsa-miR-4485-3p	MIMAT0019019	UAACGGCCGCGGUACCCUAA
hsa-miR-4488	MIMAT0019022	AGGGGGCGGGCUCCGGCG
hsa-miR-548al	MIMAT0019024	AACGGCAAUGACUUUUGUACCA
hsa-miR-4516	MIMAT0019053	GGGAGAAGGGUCGGGGC
hsa-miR-4521	MIMAT0019058	GCUAAGGAAGUCCUGUGCUCAG
hsa-miR-1269b	MIMAT0019059	CUGGACUGAGCCAUGCUACUGG
hsa-miR-4524a-5p	MIMAT0019062	AUAGCAGCAUGAACCUGUCUCA
hsa-miR-4531	MIMAT0019070	AUGGAGAAGGCUUCUGA
hsa-miR-4532	MIMAT0019071	CCCCGGGGAGCCCGGCG
hsa-miR-378i	MIMAT0019074	ACUGGACUAGGAGUCAGAAGG
hsa-miR-4536-5p	MIMAT0019078	UGUGGUAGAUAUAUGCACGAU
hsa-miR-3140-5p	MIMAT0019204	ACCUGAAUUACCAAAAGCUUU
hsa-miR-4647	MIMAT0019709	GAAGAUGGUGCUGUGCUGAGGAA
hsa-miR-219b-3p	MIMAT0019748	AGAAUUGCGUUUGGACAAUCAGU
hsa-miR-4707-5p	MIMAT0019807	GCCCCGGCGCGGGCGGGUUCUGG
hsa-miR-4707-3p	MIMAT0019808	AGCCCGCCCCAGCCGAGGUUCU
hsa-miR-4741	MIMAT0019871	CGGGCUGUCCGGAGGGGUCGGCU
hsa-miR-371b-5p	MIMAT0019892	ACUCAAAAGAUGGCGGCACUUU
hsa-miR-4755-5p	MIMAT0019895	UUUCCCUUCAGAGCCUGGCUUU
hsa-miR-499b-5p	MIMAT0019897	ACAGACUUGCUGUGAUGUUCA
hsa-miR-499b-3p	MIMAT0019898	AACAUCACUGCAAGUCUUAACA
hsa-miR-1245b-5p	MIMAT0019950	UAGGCCUUUAGAUCACUUAAA
hsa-miR-1245b-3p	MIMAT0019951	UCAGAUGAUCUAAAGGCCUAUA
hsa-miR-4787-5p	MIMAT0019956	GCGGGGGUGGCGGCGGCAUCCC
hsa-miR-4787-3p	MIMAT0019957	GAUGCGCCGCCCACUGCCCCGCGC
hsa-miR-4792	MIMAT0019964	CGGUGAGCGCUCGCUGGC

Official symbol	Accession number	Target Sequence
hsa-miR-642a-3p	MIMAT0020924	AGACACAUUUGGAGAGGGAACC
hsa-miR-548ah-3p	MIMAT0020957	CAAAAACUGCAGUUACUUUUGC
hsa-miR-4536-3p	MIMAT0020959	UCGUGCAUAUAUCUACCACAU
hsa-miR-5001-5p	MIMAT0021021	AGGGCUGGACUCAGCGGCGGAGCU
hsa-miR-5001-3p	MIMAT0021022	UUCUGCCUCUGUCCAGGUCCUU
hsa-miR-5010-5p	MIMAT0021043	AGGGGGAUGGCAGAGCAAAAUU
hsa-miR-5010-3p	MIMAT0021044	UUUUGUGUCUCCCAUUCCCCAG
hsa-miR-5196-5p	MIMAT0021128	AGGGAAGGGGACGAGGGUUGGG
hsa-miR-5196-3p	MIMAT0021129	UCAUCCUCGUCUCCCUCCCAG
hsa-miR-548ar-5p	MIMAT0022265	AAAAGUAAUUGCAGUUUUUGC
hsa-miR-548ar-3p	MIMAT0022266	UAAAACUGCAGUUAUUUUUGC
hsa-miR-664b-5p	MIMAT0022271	UGGGCUAAGGGAGAUGAUUGGGUA
hsa-miR-664b-3p	MIMAT0022272	UUCAUUUGCCUCCCAGCCUACA
hsa-miR-548av-3p	MIMAT0022304	AAAACUGCAGUUACUUUUGC
hsa-miR-197-5p	MIMAT0022691	CGGGUAGAGAGGGCAGUGGGAGG
hsa-miR-211-3p	MIMAT0022694	GCAGGGACAGCAAAGGGGUGC
hsa-miR-301a-5p	MIMAT0022696	GCUCUGACUUUAUUGCACUACU
hsa-miR-382-3p	MIMAT0022697	AAUCAUUCACGGACAACACUU
hsa-miR-345-3p	MIMAT0022698	GCCCUGAACGAGGGGUCUGGAG
hsa-miR-450a-1-3p	MIMAT0022700	AUUGGGAACAUUUUGCAUGUAU
hsa-miR-506-5p	MIMAT0022701	UAUUCAGGAAGGUGUUACUUAA
hsa-miR-514a-5p	MIMAT0022702	UACUCUGGAGAGUGACAAUCAUG
hsa-miR-539-3p	MIMAT0022705	AUCAUACAAGGACAAUUUCUUU
hsa-miR-561-5p	MIMAT0022706	AUCAAGGAUCUUAAACUUUGCC
hsa-miR-570-5p	MIMAT0022707	AAAGGUAAUUGCAGUUUUUCCC
hsa-miR-584-3p	MIMAT0022708	UCAGUUCCAGGCCAACCAGGCU
hsa-miR-652-5p	MIMAT0022709	CAACCCUAGGAGAGGGUGCCAUUCA
hsa-miR-660-3p	MIMAT0022711	ACCUCCUGUGUGCAUGGAUUA
hsa-miR-1271-3p	MIMAT0022712	AGUGCCUGCUAUGUGCCAGGCA
hsa-miR-1185-2-3p	MIMAT0022713	AUAUACAGGGGGAGACUCUCAU
hsa-miR-766-5p	MIMAT0022714	AGGAGGAAUUGGUGCUGGUCUU
hsa-miR-873-3p	MIMAT0022717	GGAGACUGAUGAGUUCCCGGGA
hsa-miR-1285-5p	MIMAT0022719	GAUCUCACUUUGUUGCCCAGG
hsa-miR-1304-3p	MIMAT0022720	UCUCACUGUAGCCUCGAACCCC
hsa-miR-548h-3p	MIMAT0022723	CAAAAACCGCAAUUACUUUUGCA

Official symbol	Accession number	Target Sequence
hsa-miR-1306-5p	MIMAT0022726	CCACCUCCCCUGCAAACGUCCA
hsa-miR-1307-5p	MIMAT0022727	UCGACCGGACCUCGACCGGCU
hsa-miR-513c-3p	MIMAT0022728	UAAAUUUCACCUUUCUGAGAAGA
hsa-miR-548t-3p	MIMAT0022730	AAAAACCACAAUUACUUUUGCACCA
hsa-miR-548o-5p	MIMAT0022738	AAAAGUAAUUGCGGUUUUUGCC
hsa-miR-548am-5p	MIMAT0022740	AAAAGUAAUUGCGGUUUUUGCC
hsa-miR-365b-5p	MIMAT0022833	AGGGACUUUCAGGGGCAGCUGU
hsa-miR-365b-3p	MIMAT0022834	UAAUGCCCCUAAAAAUCCUUAU
hsa-miR-1185-1-3p	MIMAT0022838	AUAUACAGGGGGAGACUCUUAU
hsa-miR-3190-3p	MIMAT0022839	UGUGGAAGGUAGACGGCCAGAGA
hsa-miR-98-3p	MIMAT0022842	CUAUACAACUUACUACUUUCCC
hsa-miR-376c-5p	MIMAT0022861	GGUGGAUAUUCCUUCUAUGUU
hsa-miR-381-5p	MIMAT0022862	AGCGAGGUUGCCCUUUGUAUAU
hsa-miR-495-5p	MIMAT0022924	GAAGUUGCCCAUGUUAUUUUCG
hsa-miR-503-3p	MIMAT0022925	GGGGUAUUGUUUCCGCUGCCAGG
hsa-miR-376a-2-5p	MIMAT0022928	GGUAGAUUUUCCUUCUAUGGU
hsa-miR-758-5p	MIMAT0022929	GAUGGUUGACCAGAGAGCACAC
hsa-miR-6503-5p	MIMAT0025462	AGGUCUGCAUUCAAAUCCCCAGA
hsa-miR-6503-3p	MIMAT0025463	GGGACUAGGAUGCAGACCUCC
hsa-miR-6511a-5p	MIMAT0025478	CAGGCAGAAGUGGGGCUGACAGG
hsa-miR-6511a-3p	MIMAT0025479	CCUCACCAUCCCUUCUGCCUGC
hsa-miR-6720-3p	MIMAT0025851	CGCGCCUGCAGGAACUGGUAGA
hsa-miR-6721-5p	MIMAT0025852	UGGGCAGGGGCUUAUUGUAGGAG
hsa-miR-6724-5p	MIMAT0025856	CUGGGCCCGCGGCGGGCGUGGGG
hsa-miR-210-5p	MIMAT0026475	AGCCCCUGCCCACCGCACACUG
hsa-miR-128-1-5p	MIMAT0026477	CGGGGCCGUAGCACUGUCUGAGA
hsa-miR-133a-5p	MIMAT0026478	AGCUGGUAAAAUGGAACCAAAU
hsa-miR-152-5p	MIMAT0026479	AGGUUCUGUGAUACACUCCGACU
hsa-miR-134-3p	MIMAT0026481	CCUGUGGGCCACCUAGUCACCAA
hsa-miR-190a-3p	MIMAT0026482	CUAUAUAUCAAACAUAUUCCU
hsa-miR-370-5p	MIMAT0026483	CAGGUCACGUCUCUGCAGUUAC
hsa-miR-328-5p	MIMAT0026486	GGGGGGGCAGGAGGGGCUCAGGG
hsa-miR-433-5p	MIMAT0026554	UACGGUGAGCCUGUCAUUAUUC
hsa-miR-329-5p	MIMAT0026555	GAGGUUUUCUGGGUUUCUGUUUC
hsa-miR-494-5p	MIMAT0026607	AGGUUGUCCGUGUUGUCUUCUCU

Official symbol	Accession number	Target Sequence
hsa-miR-181d-3p	MIMAT0026608	CCACCGGGGGAUGAAUGUCAC
hsa-miR-504-3p	MIMAT0026612	GGGAGUGCAGGGCAGGGUUUC
hsa-miR-510-3p	MIMAT0026613	AUUGAAACCUCUAAGAGUGGA
hsa-miR-487b-5p	MIMAT0026614	GUGGUUAUCCCUGUCCUGUUCG
hsa-miR-579-5p	MIMAT0026616	UCGCGGUUUGUGCCAGAUGACG
hsa-miR-627-3p	MIMAT0026623	UCUUUUCUUUGAGACUCACU
hsa-miR-651-3p	MIMAT0026624	AAAGGAAAGUGUAUCCUAAAAG
hsa-miR-1296-3p	MIMAT0026637	GAGUGGGGCUUCGACCCUAACC
hsa-miR-874-5p	MIMAT0026718	CGGCCCCACGCACCAGGGUAAGA
hsa-miR-887-5p	MIMAT0026720	CUUGGGAGCCCUGUUAGACUC
hsa-miR-208b-5p	MIMAT0026722	AAGCUUUUUGCUCGAAUUAUGU
hsa-miR-942-3p	MIMAT0026734	CACAUGGCCGAAACAGAGAAGU
hsa-miR-548e-5p	MIMAT0026736	CAAAAGCAAUCGCGGUUUUUGC
hsa-miR-548j-3p	MIMAT0026737	CAAAAACUGCAUUACUUUUGC
hsa-miR-1287-3p	MIMAT0026738	CUCUAGCCACAGAUGCAGUGAU
hsa-miR-1908-3p	MIMAT0026916	CCGGCCGCCGGCUCCGCCCCG
hsa-miR-1910-3p	MIMAT0026917	GAGGCAGAAGCAGGAUGACA
hsa-miR-6728-5p	MIMAT0027357	UUGGGAUGGUAGGACCAGAGGGG
hsa-miR-6732-3p	MIMAT0027366	UAACCCUGUCCUCUCCCUCCCAG
hsa-miR-450a-2-3p	MIMAT0031074	AUUGGGGACAUUUUGCAUUCAU
hsa-miR-128-2-5p	MIMAT0031095	GGGGGCCGAUACACUGUACGAGA
hsa-miR-7975	MIMAT0031178	AUCCUAGUCACGGCACCA
hsa-miR-203a-5p	MIMAT0031890	AGUGGUUCUUAACAGUUCAACAGUU
hsa-miR-1-5p	MIMAT0031892	ACAUACUUCUUUAUAUGCCCAU
hsa-miR-181b-2-3p	MIMAT0031893	CUCACUGAUCAAUGAAUGCA
hsa-miR-301b-5p	MIMAT0032026	GCUCUGACGAGGUUGCACUACU
hsa-miR-1249-5p	MIMAT0032029	AGGAGGGAGGAGAUGGGCCAAGUU
**Non-Mammalian Spike In miRNA probes**		
ath-miR159a	MIMAT0000177	UUUGGAUUGAAGGGAGCUCUA
cel-miR-248	MIMAT0000304	AUACACGUGCACGGAUAACGCUCA
cel-miR-254	MIMAT0000310	UGCAAAUCUUUCGCGACUGUAGG
osa-miR414	MIMAT0001330	UCAUCCUCAUCAUCAUCGUCC
osa-miR442	MIMAT0001605	UGACGUGUAAAUUGCGAGACGAAU
**Internal Reference Genes**		

Official symbol	Accession number	Target Sequence
ACTB	NM_001101.2	TGCAGAAGGAGATCACTGCCCTGGCACCCAGCACAATGAAGATCAAGATCATTGCTCCTCCTGAGCGCAAGTACTCCGTGTGGATCGGCGGCTCCATCCT
B2M	NM_004048.2	CGGGCATTCCTGAAGCTGACAGCATTCGGGCCGAGATGTCTCGCTCCGTGGCCTTAGCTGTGCTCGCGCTACTCTCTCTTTCTGGCCTGGAGGCTATCCA
GAPDH	NM_002046.3	TCCTCCTGTTCGACAGTCAGCCGCATCTTCTTTTGCGTCGCCAGCCGAGCCACATCGCTCAGACACCATGGGGAAGGTGAAGGTCGGAGTCAACGGATTT
RPL19	NM_000981.3	CCAATGCCCGAATGCCAGAGAAGGTCACATGGATGAGGAGAATGAGGATTTTGCGCCGGCTGCTCAGAAGATACCGTGAATCTAAGAAGATCGATCGCCA
RPLP0	NM_001002.3	CGAAATGTTTCATTGTGGGAGCAGACAATGTGGGCTCCAAGCAGATGCAGCAGATCCGCATGTCCCTTCGCGGGAAGGCTGTGGTGCTGATGGGCAAGAA

eTable 2. Difference in expression levels of 91 miRNAs between children with and without PPCS across the three timepoints*

miRNA	Unadjusted P-value	Adjusted P-value (Bonferroni Correction)	Adjusted P-value (False Discovery Rate Correction)
hsa-miR-548y	0.00001	0.00091	0.00015
hsa-miR-585-3p	0.00001	0.00091	0.00015
hsa-miR-378h	0.00001	0.00091	0.00015
hsa-miR-1323	0.00001	0.00091	0.00015
hsa-miR-183-5p	0.00001	0.00091	0.00015
hsa-miR-199a-3p+hsa-miR-199b-3p	0.00001	0.00091	0.00015
hsa-miR-301a-5p	0.00011	0.01039	0.00148
hsa-miR-626	0.00021	0.0195	0.00244
hsa-miR-203a-5p	0.00032	0.0287	0.00319
hsa-miR-888-5p	0.00057	0.05178	0.00518
hsa-miR-548e-5p	0.00084	0.07659	0.00696
hsa-miR-200a-3p	0.00104	0.0947	0.00789
hsa-miR-95-3p	0.00144	0.13136	0.0101
hsa-miR-639	0.00864	0.78652	0.05618
hsa-miR-1262	0.01254	1	0.07209
hsa-miR-342-3p	0.01267	1	0.07209
hsa-miR-922	0.01737	1	0.09088
hsa-miR-1305	0.01798	1	0.09088
hsa-miR-223-3p	0.02244	1	0.10531
hsa-miR-378i	0.02315	1	0.10531
hsa-miR-34a-5p	0.031	1	0.13432
hsa-miR-1290	0.03475	1	0.14376
hsa-miR-423-5p	0.04459	1	0.17063
hsa-miR-191-5p	0.045	1	0.17063
hsa-miR-30e-5p	0.04875	1	0.17064
hsa-miR-125b-5p	0.05088	1	0.17064
hsa-miR-664a-3p	0.0516	1	0.17064
hsa-miR-106b-5p	0.0525	1	0.17064
hsa-miR-1285-5p	0.06022	1	0.18896
hsa-miR-29a-3p	0.072	1	0.2184
hsa-miR-93-5p	0.08365	1	0.24555
hsa-miR-28-5p	0.09366	1	0.26342
hsa-let-7b-5p	0.09553	1	0.26342
hsa-miR-148a-3p	0.10501	1	0.28106
hsa-miR-4516	0.12467	1	0.32414
hsa-miR-140-5p	0.14174	1	0.35026

hsa-let-7a-5p	0.14241	1	0.35026
hsa-miR-1973	0.14714	1	0.35235
hsa-miR-181a-5p	0.1618	1	0.37712
hsa-miR-361-5p	0.16577	1	0.37712
hsa-miR-26b-5p	0.17017	1	0.37769
hsa-miR-598-3p	0.18834	1	0.40195
hsa-miR-186-5p	0.18993	1	0.40195
hsa-miR-29b-3p	0.21236	1	0.42621
hsa-miR-612	0.21284	1	0.42621
hsa-miR-15b-5p	0.22252	1	0.42621
hsa-miR-451a	0.2228	1	0.42621
hsa-miR-23a-3p	0.22482	1	0.42621
hsa-miR-4454+hsa-miR-7975	0.23355	1	0.43373
hsa-miR-1972	0.24038	1	0.43749
hsa-miR-20a-5p+hsa-miR-20b-5p	0.24593	1	0.43881
hsa-miR-142-3p	0.26561	1	0.46481
hsa-miR-16-5p	0.27645	1	0.47467
hsa-miR-200c-3p	0.29362	1	0.4948
hsa-miR-2116-5p	0.31494	1	0.51947
hsa-miR-221-3p	0.31967	1	0.51947
hsa-miR-148b-3p	0.33743	1	0.52139
hsa-miR-98-5p	0.34034	1	0.52139
hsa-miR-200b-3p	0.34192	1	0.52139
hsa-miR-150-5p	0.34377	1	0.52139
hsa-let-7c-5p	0.36431	1	0.53669
hsa-let-7d-5p	0.36566	1	0.53669
hsa-miR-363-3p	0.39772	1	0.57161
hsa-miR-30d-5p	0.40201	1	0.57161
hsa-miR-205-5p	0.41466	1	0.58052
hsa-miR-106a-5p+hsa-miR-17-5p	0.44389	1	0.61203
hsa-miR-25-3p	0.47458	1	0.63612
hsa-miR-24-3p	0.47534	1	0.63612
hsa-miR-6721-5p	0.51801	1	0.67833
hsa-let-7i-5p	0.52179	1	0.67833
hsa-miR-21-5p	0.53613	1	0.68715
hsa-miR-23b-3p	0.57456	1	0.72618
hsa-miR-4284	0.64314	1	0.79069
hsa-miR-203a-3p	0.65019	1	0.79069
hsa-miR-107	0.65167	1	0.79069
hsa-miR-141-3p	0.66772	1	0.79261
hsa-miR-579-3p	0.67067	1	0.79261
hsa-miR-1246	0.7334	1	0.85564
hsa-miR-15a-5p	0.78385	1	0.88569

hsa-miR-630	0.79449	1	0.88569
hsa-let-7g-5p	0.80383	1	0.88569
hsa-miR-130a-3p	0.81686	1	0.88569
hsa-miR-222-3p	0.8232	1	0.88569
hsa-miR-374a-5p	0.82576	1	0.88569
hsa-miR-1260a	0.8273	1	0.88569
hsa-miR-26a-5p	0.9004	1	0.94515
hsa-miR-99a-5p	0.9036	1	0.94515
hsa-miR-873-3p	0.91541	1	0.94661
hsa-miR-4286	0.96223	1	0.98385
hsa-miR-22-3p	0.97939	1	0.98423
hsa-miR-575	0.98423	1	0.98423

* Results based on longitudinal models with main effects of time and presence of PPCS

miRNA ID	Gene Symbol	Validated miRTarBase ID
hsa-miR-1323	AAGAB	MIRT762523
hsa-miR-1323	ADCYAP1	MIRT639087
hsa-miR-1323	ARL6IP1	MIRT762524
hsa-miR-1323	ATP8B3	MIRT541672
hsa-miR-1323	BIRC5	MIRT509853
hsa-miR-1323	CDH1	MIRT438026
hsa-miR-1323	CLIC4	MIRT065625
hsa-miR-1323	DAB2	MIRT478462
hsa-miR-1323	DCP1A	MIRT783741
hsa-miR-1323	DDX6	MIRT538114
hsa-miR-1323	EIF4EBP2	MIRT762526
hsa-miR-1323	EXPH5	MIRT762527
hsa-miR-1323	GJD2	MIRT657867
hsa-miR-1323	GNPTAB	MIRT557579
hsa-miR-1323	HMGN2	MIRT762530
hsa-miR-1323	HNRNPC	MIRT762531
hsa-miR-1323	KLF10	MIRT172130
hsa-miR-1323	MAMLD1	MIRT762532
hsa-miR-1323	MOCOS	MIRT531968
hsa-miR-1323	MTA1	MIRT694367
hsa-miR-1323	MTMR9	MIRT783742
hsa-miR-1323	PEG10	MIRT547156
hsa-miR-1323	PLEKHG7	MIRT610948
hsa-miR-1323	PTBP3	MIRT630580
hsa-miR-1323	RESF1	MIRT664056
hsa-miR-1323	RLIM	MIRT534711
hsa-miR-1323	RNF115	MIRT641958
hsa-miR-1323	SCIN	MIRT504629
hsa-miR-1323	SH3PXD2A	MIRT653869
hsa-miR-1323	SPRY1	MIRT539973
hsa-miR-1323	TERF2IP	MIRT490142
hsa-miR-1323	TRAPPC8	MIRT652225
hsa-miR-1323	YIPF4	MIRT443743
hsa-miR-1323	ZNF585B	MIRT525963
hsa-miR-1323	ZNF772	MIRT545799
hsa-miR-183-5p	ABCC10	MIRT047155
hsa-miR-183-5p	ABRAXAS2	MIRT047107
hsa-miR-183-5p	ACP6	MIRT645996
hsa-miR-183-5p	ARHGAP21	MIRT047126
hsa-miR-183-5p	ARHGEF5	MIRT047144
hsa-miR-183-5p	BRWD1	MIRT705229
hsa-miR-183-5p	BTAF1	MIRT047143

hsa-miR-183-5p	C18orf25	MIRT047073
hsa-miR-183-5p	C5orf24	MIRT047162
hsa-miR-183-5p	CAPZA1	MIRT763160
hsa-miR-183-5p	CCNB1	MIRT047127
hsa-miR-183-5p	CCND1	MIRT024995
hsa-miR-183-5p	CELF1	MIRT572175
hsa-miR-183-5p	CR1	MIRT047066
hsa-miR-183-5p	CSNK2A1	MIRT047136
hsa-miR-183-5p	CYB5D2	MIRT047145
hsa-miR-183-5p	DBT	MIRT676869
hsa-miR-183-5p	DDAH1	MIRT558453
hsa-miR-183-5p	DKK3	MIRT053048
hsa-miR-183-5p	DNTTIP2	MIRT763161
hsa-miR-183-5p	DOCK1	MIRT047082
hsa-miR-183-5p	DSN1	MIRT763162
hsa-miR-183-5p	EGR1	MIRT006540
hsa-miR-183-5p	EIF4A3	MIRT047149
hsa-miR-183-5p	EIF4EBP2	MIRT658779
hsa-miR-183-5p	ELAC2	MIRT047119
hsa-miR-183-5p	ERBIN	MIRT703885
hsa-miR-183-5p	ERC1	MIRT024998
hsa-miR-183-5p	ERG28	MIRT047164
hsa-miR-183-5p	FAT2	MIRT047135
hsa-miR-183-5p	FBXO32	MIRT240100
hsa-miR-183-5p	FEN1	MIRT047080
hsa-miR-183-5p	FNTB	MIRT047154
hsa-miR-183-5p	FOXN2	MIRT047161
hsa-miR-183-5p	GLO1	MIRT047098
hsa-miR-183-5p	GLUL	MIRT025018
hsa-miR-183-5p	GNL3	MIRT047156
hsa-miR-183-5p	GP2	MIRT672296
hsa-miR-183-5p	GSPT1	MIRT047129
hsa-miR-183-5p	GUCD1	MIRT047169
hsa-miR-183-5p	HARS1	MIRT047138
hsa-miR-183-5p	HIPK2	MIRT047166
hsa-miR-183-5p	HSP90AA1	MIRT047124
hsa-miR-183-5p	HSPA13	MIRT547871
hsa-miR-183-5p	HYOU1	MIRT047102
hsa-miR-183-5p	IGF1R	MIRT025009
hsa-miR-183-5p	INSIG1	MIRT047148
hsa-miR-183-5p	ITGA8	MIRT047150
hsa-miR-183-5p	ITPKB	MIRT047075
hsa-miR-183-5p	KCNS2	MIRT719182

hsa-miR-183-5p	KCTD15	MIRT513849
hsa-miR-183-5p	KIF2A	MIRT004016
hsa-miR-183-5p	KLHL23	MIRT047139
hsa-miR-183-5p	KLRD1	MIRT527794
hsa-miR-183-5p	KNTC1	MIRT047167
hsa-miR-183-5p	KY	MIRT526274
hsa-miR-183-5p	LARS1	MIRT047117
hsa-miR-183-5p	LENG8	MIRT047171
hsa-miR-183-5p	LRP6	MIRT052934
hsa-miR-183-5p	LRRC58	MIRT354492
hsa-miR-183-5p	LYPLA1	MIRT047071
hsa-miR-183-5p	MARF1	MIRT047170
hsa-miR-183-5p	MBD4	MIRT501918
hsa-miR-183-5p	MCM4	MIRT047147
hsa-miR-183-5p	MDM4	MIRT334810
hsa-miR-183-5p	METTL2B	MIRT664085
hsa-miR-183-5p	MIPOL1	MIRT723431
hsa-miR-183-5p	MORF4L1	MIRT541184
hsa-miR-183-5p	MRTFA	MIRT047070
hsa-miR-183-5p	MRTFB	MIRT566790
hsa-miR-183-5p	MSH2	MIRT047085
hsa-miR-183-5p	MTUS1	MIRT047094
hsa-miR-183-5p	MYBBP1A	MIRT047122
hsa-miR-183-5p	MYCBP2	MIRT047123
hsa-miR-183-5p	MYO10	MIRT047146
hsa-miR-183-5p	NEO1	MIRT047083
hsa-miR-183-5p	NOTCH2	MIRT025026
hsa-miR-183-5p	NR3C1	MIRT378074
hsa-miR-183-5p	P4HB	MIRT047151
hsa-miR-183-5p	PAK4	MIRT642243
hsa-miR-183-5p	PDP2	MIRT632602
hsa-miR-183-5p	PEAK1	MIRT025011
hsa-miR-183-5p	PFKFB3	MIRT790135
hsa-miR-183-5p	PGM2L1	MIRT712309
hsa-miR-183-5p	PITPNM3	MIRT738181
hsa-miR-183-5p	PLEKHB2	MIRT535164
hsa-miR-183-5p	PLEKHM1	MIRT047158
hsa-miR-183-5p	POGLUT3	MIRT047165
hsa-miR-183-5p	POLH	MIRT047103
hsa-miR-183-5p	PPRC1	MIRT047060
hsa-miR-183-5p	PREB	MIRT047099
hsa-miR-183-5p	PSMD13	MIRT047134
hsa-miR-183-5p	PTP4A2	MIRT047091

hsa-miR-183-5p	PTPN11	MIRT047153
hsa-miR-183-5p	RAB5B	MIRT047065
hsa-miR-183-5p	RALGDS	MIRT047157
hsa-miR-183-5p	RDH11	MIRT047120
hsa-miR-183-5p	REV3L	MIRT025031
hsa-miR-183-5p	RIF1	MIRT047132
hsa-miR-183-5p	RNF41	MIRT025017
hsa-miR-183-5p	RPAP2	MIRT047058
hsa-miR-183-5p	RPL7L1	MIRT503543
hsa-miR-183-5p	RTN4	MIRT024991
hsa-miR-183-5p	SENP1	MIRT025028
hsa-miR-183-5p	SHISA2	MIRT047100
hsa-miR-183-5p	SLC18B1	MIRT570108
hsa-miR-183-5p	SLC25A25	MIRT467850
hsa-miR-183-5p	SLC31A1	MIRT047052
hsa-miR-183-5p	SLC35E2B	MIRT645096
hsa-miR-183-5p	SMG1	MIRT467529
hsa-miR-183-5p	SOCS6	MIRT735272
hsa-miR-183-5p	SRSF10	MIRT047131
hsa-miR-183-5p	STK33	MIRT025022
hsa-miR-183-5p	SUCO	MIRT485071
hsa-miR-183-5p	TERF2	MIRT630047
hsa-miR-183-5p	THOC2	MIRT047168
hsa-miR-183-5p	TMED7	MIRT510657
hsa-miR-183-5p	TMEM170B	MIRT565335
hsa-miR-183-5p	TMEM245	MIRT047061
hsa-miR-183-5p	TNRC6B	MIRT025025
hsa-miR-183-5p	TPRG1L	MIRT047125
hsa-miR-183-5p	TRAF4	MIRT047104
hsa-miR-183-5p	TRIM37	MIRT047093
hsa-miR-183-5p	TRIM71	MIRT533470
hsa-miR-183-5p	TRO	MIRT047116
hsa-miR-183-5p	TSKU	MIRT500671
hsa-miR-183-5p	TSR1	MIRT047141
hsa-miR-183-5p	UBP1	MIRT025006
hsa-miR-183-5p	UBXN7	MIRT047055
hsa-miR-183-5p	UCHL3	MIRT047087
hsa-miR-183-5p	UQCRC1	MIRT047130
hsa-miR-183-5p	USP19	MIRT047096
hsa-miR-183-5p	USP22	MIRT047140
hsa-miR-183-5p	USP8	MIRT047051
hsa-miR-183-5p	WDR26	MIRT271532
hsa-miR-183-5p	WDR33	MIRT723492

hsa-miR-183-5p	WDR53	MIRT047121
hsa-miR-183-5p	YY1	MIRT533067
hsa-miR-183-5p	ZC3H4	MIRT025021
hsa-miR-183-5p	ZFAT	MIRT047133
hsa-miR-183-5p	ZFHX4	MIRT047086
hsa-miR-183-5p	ZFPM1	MIRT437880
hsa-miR-183-5p	ZNF24	MIRT708282
hsa-miR-183-5p	ZNF621	MIRT619743
hsa-miR-199a-3p_hsa-miR-199b-3p	CCDC80	MIRT696977
hsa-miR-199a-3p_hsa-miR-199b-3p	CCNL1	MIRT006586
hsa-miR-199a-3p_hsa-miR-199b-3p	CSNK1A1	MIRT558557
hsa-miR-199a-3p_hsa-miR-199b-3p	FGF2	MIRT557866
hsa-miR-199a-3p_hsa-miR-199b-3p	FLT1	MIRT735200
hsa-miR-199a-3p_hsa-miR-199b-3p	FOXA2	MIRT735287
hsa-miR-199a-3p_hsa-miR-199b-3p	HGF	MIRT054477
hsa-miR-199a-3p_hsa-miR-199b-3p	KDR	MIRT735201
hsa-miR-199a-3p_hsa-miR-199b-3p	LIN54	MIRT474145
hsa-miR-199a-3p_hsa-miR-199b-3p	MAPK9	MIRT003982
hsa-miR-199a-3p_hsa-miR-199b-3p	MET	MIRT001969
hsa-miR-199a-3p_hsa-miR-199b-3p	MTOR	MIRT026023
hsa-miR-199a-3p_hsa-miR-199b-3p	PAK4	MIRT733785
hsa-miR-199a-3p_hsa-miR-199b-3p	SMYD4	MIRT763625
hsa-miR-199a-3p_hsa-miR-199b-3p	SOD2	MIRT442903
hsa-miR-199a-3p_hsa-miR-199b-3p	TFAM	MIRT731449
hsa-miR-199a-3p_hsa-miR-199b-3p	TSPAN3	MIRT553306
hsa-miR-199a-3p_hsa-miR-199b-3p	ZXDB	MIRT462875
hsa-miR-199a-3p_hsa-miR-199b-3p	CCDC80	MIRT696976
hsa-miR-199a-3p_hsa-miR-199b-3p	CSNK1A1	MIRT558556
hsa-miR-199a-3p_hsa-miR-199b-3p	FGF2	MIRT557867
hsa-miR-199a-3p_hsa-miR-199b-3p	LIN54	MIRT474143
hsa-miR-199a-3p_hsa-miR-199b-3p	PAK4	MIRT733786
hsa-miR-199a-3p_hsa-miR-199b-3p	SMYD4	MIRT763636
hsa-miR-199a-3p_hsa-miR-199b-3p	SOD2	MIRT442902
hsa-miR-199a-3p_hsa-miR-199b-3p	TSPAN3	MIRT553305
hsa-miR-199a-3p_hsa-miR-199b-3p	ZXDB	MIRT462874
hsa-miR-200a-3p	BICRAL	MIRT710918
hsa-miR-200a-3p	EGFR	MIRT735444
hsa-miR-200a-3p	ERBIN	MIRT020347
hsa-miR-200a-3p	GATA6	MIRT004006
hsa-miR-200a-3p	HMGB1	MIRT735283
hsa-miR-200a-3p	KEAP1	MIRT006704
hsa-miR-200a-3p	KIAA1549L	MIRT573383
hsa-miR-200a-3p	MAPK14	MIRT006423

hsa-miR-200a-3p	OGT	MIRT176071
hsa-miR-200a-3p	PSMD2	MIRT004400
hsa-miR-200a-3p	RBM28	MIRT696748
hsa-miR-200a-3p	RUSF1	MIRT738760
hsa-miR-200a-3p	SLC35D1	MIRT554194
hsa-miR-200a-3p	SMAD2	MIRT007290
hsa-miR-200a-3p	TFAM	MIRT053166
hsa-miR-200a-3p	ZEB1	MIRT002480
hsa-miR-203a-5p	CACNA1A	MIRT784133
hsa-miR-203a-5p	COX20	MIRT515224
hsa-miR-203a-5p	CRKL	MIRT738806
hsa-miR-203a-5p	DOCK4	MIRT524029
hsa-miR-203a-5p	MMP2	MIRT734404
hsa-miR-203a-5p	MYO10	MIRT656037
hsa-miR-203a-5p	NWD1	MIRT707118
hsa-miR-203a-5p	PTBP2	MIRT498462
hsa-miR-203a-5p	TSC22D2	MIRT465106
hsa-miR-203a-5p	ZDHHC9	MIRT763698
hsa-miR-203a-5p	ZNF28	MIRT643512
hsa-miR-301a-5p	CDK6	MIRT479452
hsa-miR-301a-5p	ERBB3	MIRT719394
hsa-miR-301a-5p	MXD1	MIRT556040
hsa-miR-301a-5p	PSG4	MIRT724766
hsa-miR-301a-5p	RREB1	MIRT699978
hsa-miR-301a-5p	SKIL	MIRT699517
hsa-miR-301a-5p	SPATS2	MIRT617242
hsa-miR-301a-5p	SPECC1L	MIRT739618
hsa-miR-301a-5p	ZNF544	MIRT118176
hsa-miR-378h	AKT1	MIRT543139
hsa-miR-378h	CYP20A1	MIRT529214
hsa-miR-378h	ESYT2	MIRT537576
hsa-miR-378h	HIPK3	MIRT725446
hsa-miR-378h	KCNJ6	MIRT495440
hsa-miR-378h	LGSN	MIRT692968
hsa-miR-378h	MYRF	MIRT655996
hsa-miR-378h	PRKD2	MIRT525109
hsa-miR-378h	RAP1B	MIRT554923
hsa-miR-378h	RDH11	MIRT767528
hsa-miR-378h	REST	MIRT469372
hsa-miR-378h	TGFB2	MIRT652733
hsa-miR-378h	TXNL1	MIRT520413
hsa-miR-378h	WDR33	MIRT723498
hsa-miR-378h	WTAP	MIRT564969

hsa-miR-378h	YY1	MIRT742772
hsa-miR-378h	ZNF609	MIRT463101
hsa-miR-548e-5p	CREG2	MIRT615787
hsa-miR-548e-5p	DIO2	MIRT628266
hsa-miR-548e-5p	EXOC5	MIRT548295
hsa-miR-548e-5p	GDNF	MIRT559411
hsa-miR-548e-5p	GPR180	MIRT190192
hsa-miR-548e-5p	NCOA1	MIRT655971
hsa-miR-548e-5p	NFATC3	MIRT566645
hsa-miR-548e-5p	SHOC2	MIRT561610
hsa-miR-548e-5p	STK4	MIRT534045
hsa-miR-548e-5p	STRADB	MIRT300115
hsa-miR-548y	ARL13B	MIRT525866
hsa-miR-548y	CHEK2	MIRT548900
hsa-miR-548y	GTF2H5	MIRT442839
hsa-miR-548y	MKLN1	MIRT551048
hsa-miR-548y	SVOP	MIRT499866
hsa-miR-548y	THRAP3	MIRT340674
hsa-miR-548y	TMEM241	MIRT550488
hsa-miR-548y	UHMK1	MIRT750837
hsa-miR-548y	ZMPSTE24	MIRT774579
hsa-miR-585-3p	CDC25B	MIRT751542
hsa-miR-585-3p	EIF5A2	MIRT703996
hsa-miR-585-3p	MAPK1	MIRT473718
hsa-miR-585-3p	TMEM175	MIRT719511
hsa-miR-626	GPAT4	MIRT716889
hsa-miR-626	PTGS1	MIRT717690
hsa-miR-626	RUFY2	MIRT699932
hsa-miR-626	SLC7A5	MIRT004144
hsa-miR-626	SMCR8	MIRT752623
hsa-miR-626	WASF2	MIRT776120
hsa-miR-888-5p	CDK13	MIRT720034
hsa-miR-888-5p	DCP2	MIRT615047
hsa-miR-888-5p	EDEM1	MIRT624071
hsa-miR-888-5p	MCTS1	MIRT572734
hsa-miR-888-5p	NIBAN1	MIRT507357
hsa-miR-888-5p	SLC39A9	MIRT666226
hsa-miR-95-3p	NXPH3	MIRT782990
